# Direct isolation of myofibroblasts and fibroblasts from bleomycin-injured lungs reveals their functional similarities and differences

**DOI:** 10.1186/1755-1536-6-15

**Published:** 2013-08-08

**Authors:** Taisuke Akamatsu, Yosifumi Arai, Isao Kosugi, Hideya Kawasaki, Shiori Meguro, Makiko Sakao, Kiyoshi Shibata, Takafumi Suda, Kingo Chida, Toshihide Iwashita

**Affiliations:** 1 Department of Respiratory Medicine, Hamamatsu University School of Medicine, 1-20-1, Handayama, Higashi-ku, Hamamatsu, Japan; 2 Department of Regenerative and Infectious Pathology, Hamamatsu University School of Medicine, 1-20-1, Handayama, Higashi-ku, Hamamatsu, Japan; 3 Department of Pathology, Seirei Hamamatsu General Hospital, 2-12-12 Sumiyoshi, Naka-ku, Hamamatsu, Japan; 4 Research Equipment Center, Hamamatsu University School of Medicine, 1-20-1, Handayama, Higashi-ku, Hamamatsu, Japan

**Keywords:** α-smooth muscle actin, Chemokine, Collagen, Fibroblasts, Myofibroblasts

## Abstract

**Background:**

Myofibroblasts play a crucial role in tissue repair. The functional similarities and differences between myofibroblasts and fibroblasts are not fully understood because they have not been separately isolated from a living body. The purpose of this study was to establish a method for the direct isolation of myofibroblasts and fibroblasts from injured lungs by using fluorescence-activated cell sorting and to compare their functions.

**Results:**

We demonstrated that lineage-specific cell surface markers (lin), such as CD31, CD45, CD146, EpCAM (CD326), TER119, and Lyve-1 were not expressed in myofibroblasts or fibroblasts. Fibroblasts of bleomycin-injured lungs and saline-treated lungs were shown to be enriched in lin^neg^ Sca-1^high^, and myofibroblasts of bleomycin-injured lungs were shown to be enriched in lin^neg^ Sca-1^low^ CD49e^high^. Results from *in-vitro* proliferation assays indicated *in-vitro* proliferation of fibroblasts but not myofibroblasts of bleomycin-injured lungs and of fibroblasts of saline-treated lungs. However, fibroblasts and myofibroblasts might have a low proliferative capacity *in vivo*. Analysis of genes for collagen and collagen synthesis enzymes by qRT-PCR showed that the expression levels of about half of the genes were significantly higher in fibroblasts and myofibroblasts of bleomycin-injured lungs than in fibroblasts of saline-treated lungs. By contrast, the expression levels of 8 of 11 chemokine genes of myofibroblasts were significantly lower than those of fibroblasts.

**Conclusions:**

This is the first study showing a direct isolation method of myofibroblasts and fibroblasts from injured lungs. We demonstrated functional similarities and differences between myofibroblasts and fibroblasts in terms of both their proliferative capacity and the expression levels of genes for collagen, collagen synthesis enzymes, and chemokines. Thus, this direct isolation method has great potential for obtaining useful information from myofibroblasts and fibroblasts.

## Background

When a tissue is injured, a sequence of events leads to the repair of the injured tissue. Tissue repair (wound repair) occurs in three overlapping phases, the inflammatory, proliferative, and remodeling phases [1]. In the inflammatory phase, leukocytes, such as neutrophils, lymphocytes, and monocytes, are recruited to clear the wound of dead cells. In the proliferative phase, angiogenesis and collagen deposition occurs, and the injured tissue is replaced with granulation tissue, which is composed of endothelial cells, pericytes, fibroblasts, myofibroblasts, leukocytes, and extracellular matrix. In the remodeling phase, repair of the injured tissue can be accomplished by the regeneration of parenchymal cells of the same type or replacement by fibrous extracellular matrix.

Myofibroblasts found in both the proliferative and remodeling phases play an important role in producing extracellular matrix, including collagen, and are defined as fibroblast-like cells that express α-smooth muscle actin (α-SMA) [2-10]. Analysis under an electron microscope indicated that myofibroblasts have morphological characteristics similar to those of both fibroblasts and smooth muscle cells [11]. However, except for the contractility of myofibroblasts, functional similarities and differences between fibroblasts and myofibroblasts have not been fully elucidated [12,13].

Fibroblasts in culture differentiate into α-SMA-positive myofibroblast-like cells with increased expression of type 1 collagen in the presence of transforming growth factor β (TGF-β), which is a strong profibrotic factor [14-16]. Many genes involved in the regulation of myofibroblast differentiation have been studied in models of *in-vitro* differentiation [17-20]. However, it is unclear whether cultured myofibroblast-like cells have the same nature as myofibroblasts present *in vivo* because the gene expression pattern might be altered during culture. Therefore, the direct isolation of myofibroblasts and fibroblasts from the living body is necessary for comparison of their functions. To our knowledge, no study has shown a direct isolation method of myofibroblasts and fibroblasts from injured tissue because these cells lack specific cell surface markers that distinguish them from other cells when using fluorescence-activated cell sorting (FACS).

In this study, we used a combination of cell surface markers to isolate myofibroblasts and fibroblasts from bleomycin-injured lungs in the proliferative phase. Furthermore, we found that fibroblasts of bleomycin-injured lungs and saline-treated lungs proliferated *in vitro*, while myofibroblasts of bleomycin-injured lungs did not proliferate *in vitro*. In addition, we compared the expression levels of genes for collagen, collagen synthesis enzymes, and chemokines of directly isolated myofibroblasts and fibroblasts. We found that the expression levels of genes for collagen and collagen synthesis enzymes were similar between myofibroblasts and fibroblasts of bleomycin-injured lungs, whereas the expression levels of chemokine genes were reduced in myofibroblasts. These results indicated that the function of myofibroblasts differs from that of fibroblasts in bleomycin-injured lungs.

## Results

### Characterization of the bleomycin-injury lung model

**Figure 1:**
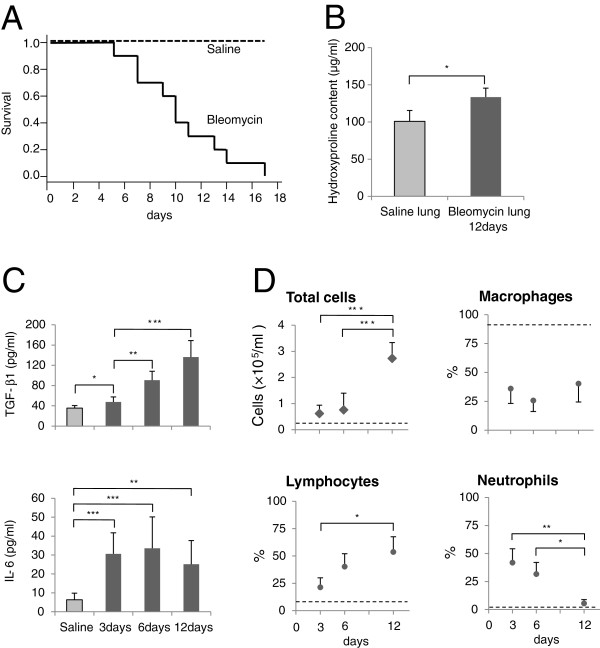
**Characterization of the bleomycin-injured lung model. (A)** Mice (solid line, *N* = 10) were intratracheally administered bleomycin. In mice treated with bleomycin, mortality was as high as 70% by day 12. **(B)** Saline-treated lungs at day 12 (*N* = 3) and bleomycin-injured lungs at day 12 (*N* = 3) were homogenized at the same time. Hydroxyproline content was higher in bleomycin-injured lungs at day 12 than in saline-treated lungs. Results represent the mean (± s.d.) of three experiments. **(C)** BAL fluid of saline-treated lungs (*N* = 3) was collected at day 12. BAL fluid of bleomycin-treated lungs was collected at days 3 (*N* = 3), 6 (*N* = 3), and 12 (*N* = 5). IL-6 and TGF-β1 concentration in BAL fluid at days 3, 6, and 12 was significantly higher than that of saline-treated lungs. Results represent the mean (± s.d.) of three or five experiments. **(D)** Total and differential cell count in BAL fluid of mice treated by bleomycin or saline (dashed line). BAL fluid of saline-treated lungs was collected at days 12 (*N* = 3). BAL fluid of bleomycin-treated lungs was collected at days 3 (*N* = 3), 6 (*N* = 3), and 12 (*N* = 5). Dashed line indicates the average of BAL fluid of saline-treated lungs. Results represent the mean (± s.d.) of three or five experiments. *, *P* < 0.05; **, *P* < 0.001; ***, *P* < 0.0001. BAL, bronchoalveolar lavage; s.d., standard deviation.

In the bleomycin-injury lung model used in this study (intratracheal administration of bleomycin 2 mg/kg to 10- to 12-week-old mice), the mortality was as high as 70% by day 12 (Figure 1A). To reduce the number of mice used for experiments, bleomycin-injured lungs of mice at day 12 were used throughout this study. The hydroxyproline content of bleomycin-injured lungs of day 12 was higher (1.3-fold higher, *P* < 0.05) than that of saline-treated lungs (Figure 1B). The content of TGF-β1 in bronchoalveolar lavage (BAL) fluid of bleomycin-injured lungs at day 12 was significantly higher than those of bleomycin-injured lungs at day 3 and saline-treated lungs (Figure 1C). These results indicated that collagen deposition had occurred concomitantly with the increased levels of TGF-β1 in bleomycin-injured lungs of day 12.

The content of IL-6, a pro-inflammatory cytokine, of bleomycin-injured lungs at day 12 was comparable to those of bleomycin-injured lungs at days 3 and 6. The percentage of neutrophils in BAL fluid of bleomycin-injured lungs at day 12 was significantly decreased compared with those of bleomycin-injured lungs at day 6, whereas the percentage of lymphocytes in BAL fluid of bleomycin-injured lungs at day 12 reached was around 50% (Figures 1C and D). These results indicated that the acute inflammatory response had ended, and the chronic inflammatory response continued in bleomycin-injured lungs at day 12.

Extensive lung fibrosis typically occurs around days 21 to 28 in the remodeling phase after intratracheal administration of bleomycin [21,22]. Thus, we hypothesized that the collagen deposition found in bleomycin-injured lungs of day 12 occurred in the proliferative phase, between the inflammatory phase and remodeling phase.

### Myofibroblasts expressed type 1 collagen in bleomycin-injured lungs

**Figure 2:**
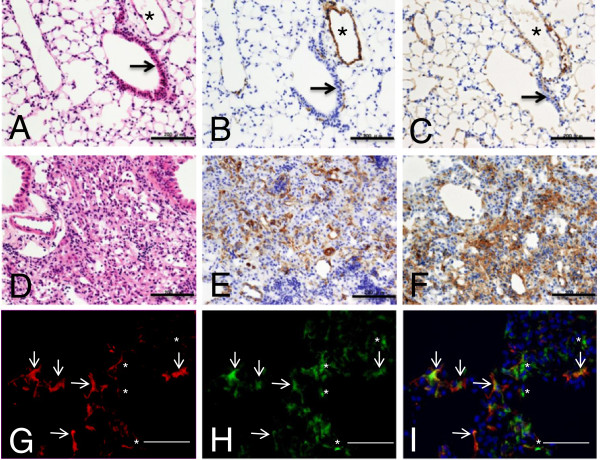
**Myofibroblasts expressed type 1 collagen in bleomycin-injured lungs.** Saline-treated lungs were fixed in formalin and examined by H & E stain **(A)** and using anti-α-SMA antibody **(B)** and anti-Col1A1 antibody **(C)**. 12 days after bleomycin had been administered, bleomycin-injured lungs fixed in formalin were examined by H & E stain **(D)** and using anti-α-SMA antibody **(E)** and anti-Col1A1 antibody **(F)**. Snap-frozen bleomycin-injured lungs at day 12 were fixed in cold acetone and examined by immunofluorescence using anti-α-SMA antibody **(G)** and anti-Col1A1 antibody **(H)**, and these latter two images were merged with nuclear stain using DAPI **(I)**. Asterisk and arrow indicate pulmonary and bronchial epithelium, respectively. In **(G)**, **(H)**, and **(I)**, arrows indicate cells that were α-SMA- and Col1A1 double-positive. The asterisk indicates Col1A1 in the interstitial space. Figures show representative results. Scale bars in **(A** to **F)** indicate 200 μm. Scale bars in (G to I) indicate 100 μm. α-SMA, α-smooth muscle actin; Col1A1, collagen 1A1, DAPI, 4´,6-diamidino-2-phenylindole.

The expression of α-SMA was examined immunohistochemically in saline-treated lungs and bleomycin-injured lungs. Except for vascular and bronchial smooth muscle cells, α-SMA-positive cells were not found in saline-treated lungs (Figure 2B). On day 12 after administration of bleomycin, α-SMA-positive myofibroblasts were found in lungs. Type 1 collagen A1 (Col1A1), which is a component of type 1 collagen, was localized primarily in the perivascular area (adventitia) in saline-treated lungs (Figure 2C). Weak immunoreactivity of Col1A1 was found in the vascular smooth muscle layer, indicating that vascular smooth muscle cells partly produce type 1 collagen, as previously reported (data not shown) [23,24]. Strong immunoreactivity of Col1A1 was detected in regions where myofibroblasts were found in bleomycin-injured lungs at day 12 (Figure 2E). Immunofluorescence analysis using antibodies against α-SMA and Col1A1 demonstrated that myofibroblasts produced collagen 1A1 in bleomycin-injured lungs at day 12 (Figures 2G,H, and I).

These results indicated that in bleomycin-injured lungs, type 1 collagen was primarily produced by myofibroblasts and perivascular fibroblasts, whereas in saline-treated lungs, type 1 collagen was primarily produced by perivascular fibroblasts.

### Lineage-specific cell surface markers were not expressed in myofibroblasts or perivascular cells

To isolate myofibroblasts and fibroblasts from bleomycin-injured lungs at day 12, many cell types should be eliminated when using FACS. Lungs are composed of many types of cell: epithelial cell adhesion molecule (EpCAM)-positive epithelial cells, CD31-positive vascular endothelial cells, lymphatic vessel endothelial hyaluronan receptor (Lyve-1)-positive lymphatic endothelial cells, TER119-positive erythrocytes, CD45-positive leukocytes, fibroblasts, myofibroblasts, pericytes, mesothelial cells, cartilage cells, and adipocytes. Adipocytes and cartilage cells can easily be excised because they are located primarily in the hilum of the lung.

Neuron-glial antigen 2 (NG2), but not α-SMA, is expressed in pericytes in mouse capillary vessels, as previously reported [25]. NG2 was specifically expressed in pericytes and vascular smooth muscle cells in lungs of NG2DsRedBAC transgenic mice (in Additional file 1: Figure S1A). Analysis by immunohistochemistry and flow cytometry showed that CD146 was expressed on the cell surface of most NG2-positive cells of these transgenic mice (in Additional file 1: Figure S1B and Additional file 1: Figure S2). Moreover, CD146 was expressed in bronchial smooth muscle cells (in Additional file 1: Figure S1C). These results demonstrate that CD146 is a lineage-specific cell surface marker of pericytes, vascular smooth muscle cells, and bronchial smooth muscle cells.

**Figure 3:**
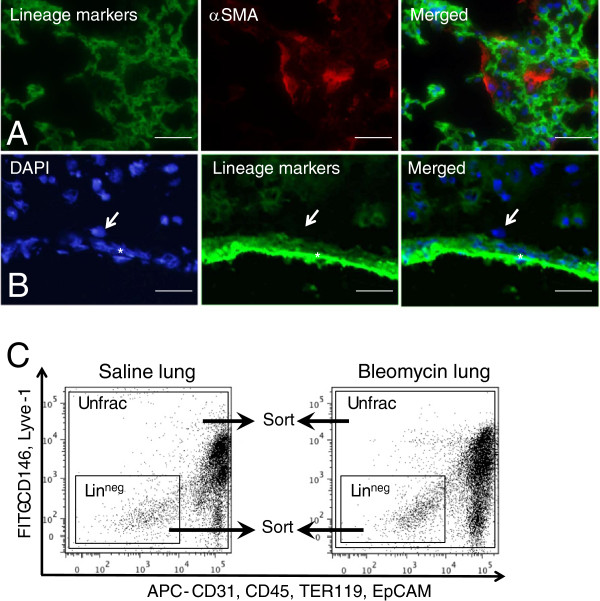
**Lineage-specific cell surface markers were not expressed in myofibroblasts or perivascular cells. (A)** Snap-frozen bleomycin-injured lungs at day 12 were fixed in acetone and examined by immunofluorescence using FITC-conjugated antibodies to lineage-specific cell surface markers (CD31, CD45, EpCAM, TER119, CD146, and Lyve-1) (left) and anti-α-SMA antibody (middle); these two images were merged with DAPI (blue in right). Figures show representative results. Scale bars indicate 100 μm. **(B)** Snap-frozen saline-treated lungs were fixed in acetone and examined by immunofluorescence with DAPI (left) and FITC-conjugated antibodies to lineage-specific cell surface markers (CD31, CD45, EpCAM, TER119, CD146, and Lyve-1) (middle); these two images were merged (right). Arrows indicate a cell that was localized in the adventitia of the pulmonary artery and that was negative for lineage-specific cell surface markers. The asterisk indicates the pulmonary artery. Figures show representative results. Scale bars indicate 50 μm. **(C)** Single lung cells, as shown in Additional file 1: Figure S3 were plotted for APC-conjugated anti-CD31, CD45, EpCAM, and TER119 antibodies vs. FITC-conjugated anti-CD146 and Lyve-1 antibodies. Unfractionated cells were sorted from the square in the figure. Lin^neg^ cells were sorted from the APC and FITC double-negative fraction, as shown in the oblong. Unfrac, unfractionated; DAPI, 4′,6-diamidino-2-phenylindole.

We used immunofluorescence analysis to demonstrate that perivascular cells and α-SMA-positive myofibroblasts were negative for lineage-specific cell surface markers (CD31, CD45, CD146, EpCAM, Lyve-1, and TER119), as shown in Figures 3A and B.

### Myofibroblasts and fibroblasts were enriched in lin^neg^ cells of saline-treated lungs and bleomycin-injured lungs

The results of our immunofluorescence analyses (Figures 3A and B) support the hypothesis that myofibroblasts and fibroblasts are enriched in the fraction that was negative for lineage-specific cell surface markers when using FACS (lin^neg^ cells) (Figure 3C and Additional file 1: Figure S3).

**Figure 4:**
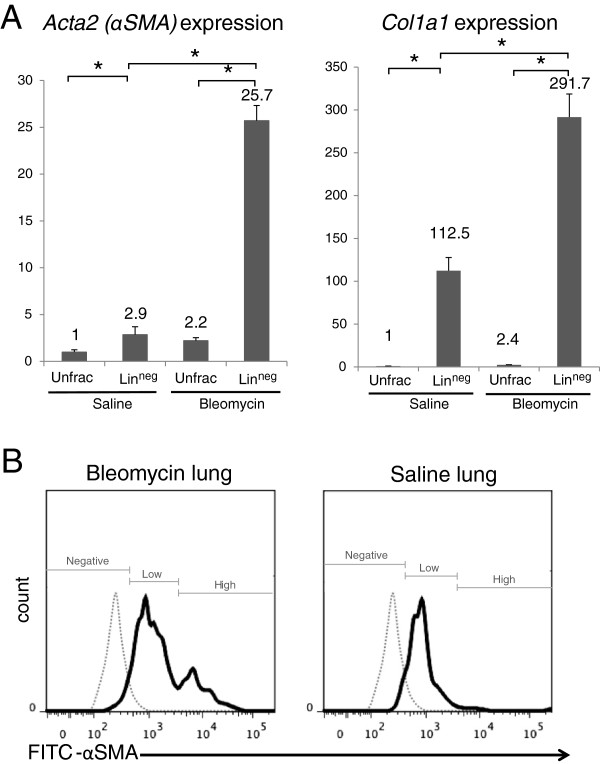
**Myofibroblasts and fibroblasts were enriched in lin**^**neg **^**cells of saline-treated lungs and bleomycin-injured lungs. (A)** Expression levels of *Acta2* and *Col1a1*mRNA were compared among unfractionated cells of saline-treated lungs, lin^neg^ cells of saline-treated lungs, unfractionated cells of bleomycin-injured lungs at day 12, and lin^neg^ cells of bleomycin-injured lungs at day 12 using qRT-PCR. Results were normalized to expression levels of unfractionated cells of saline-treated lungs. QRT-PCR was performed in triplicate using three independently prepared RNA samples. Results represent the mean (± s.d.) of three experiments. *, *P* < 0.01. **(B)** Expression levels of intracellular α-SMA were compared between lin^neg^ cells of bleomycin-injured lungs at day 12 (left) and lin^neg^ cells of saline-treated lungs (right) using FITC-conjugated anti-α-SMA antibody. As shown in Figure 2C, 10,000 sorted lin^neg^ cells were fixed in 10% formalin and then permeabilized and incubated with mouse FITC-conjugated isotype control antibody (IgG_2a_) or FITC-conjugated anti-α-SMA antibody. The fluorescence intensity of FITC-α-SMA (black line) or FITC-conjugated isotype control (gray line) of lin^neg^ cells is shown. Figures show representative results. Unfrac, unfractionated; s.d., standard deviation.

To confirm the hypothesis, gene expression levels of *Col1a1* (a fibroblast and myofibroblast marker) and *Acta2* (a gene encoding α-SMA) were compared between unfractionated cells and lin^neg^ cells, using qRT-PCR. In saline-treated lungs, *Acta2* and *Col1a1* expression levels of lin^neg^ cells were 2.9-fold and 112.5-fold higher than corresponding levels in unfractionated cells (Figure 4A). In bleomycin-injured lungs at day 12, *Acta2* and *Col1a1* expression levels of lin^neg^ cells were 11.7-fold and 121.5-fold higher than corresponding levels in unfractionated cells (Figure 4A). Quantification of vimentin (a mesenchymal cell marker) by flow cytometry showed that vimentin was expressed in most lin^neg^ cells of saline-treated lungs and bleomycin-injured lungs of day 12 (data not shown), suggesting that lin^neg^ cells did not contain epithelial cells but consisted primarily of fibroblasts and myofibroblasts. Quantification of α-SMA by flow cytometry showed that approximately 25% of lin^neg^ cells of bleomycin-injured lungs expressed high levels of α-SMA and that the remaining 75% of lin^neg^ cells of bleomycin-injured lungs at day 12 and most lin^neg^ cells of saline-treated lungs expressed low levels of α-SMA (Figure 4B).

From these findings, we concluded that myofibroblasts (α-SMA^high^*Col1a1*^*high*^) and fibroblasts (α-SMA^low^*Col1a1*^*high*^) were enriched in lin^neg^ cells of bleomycin-injured lungs of day 12 and that fibroblasts (α-SMA^low^*Col1a1*^*high*^) were enriched in lin^neg^ cells of saline-treated lungs.

### Exploring cell surface markers of myofibroblasts and fibroblasts

To investigate cell surface markers that are highly expressed in myofibroblasts, we generated cDNA of lin^neg^ cells of bleomycin-injured lungs at day 12 and performed quantitative PCR for 114 genes of cell surface markers ( in Additional file 1: Table S2), for which antibodies were commercially available for flow cytometry, and measured the cycle threshold (Ct) of each gene (Table 1). The cycle threshold is defined as the number of cycles that were required for the fluorescent signal to cross the threshold. Because a low Ct implies that the gene is expressed at high levels, gene expression levels can be estimated from the Ct [26].

**Table 1:** Exploring cell surface markers of myofibroblasts

**Gene**	**ΔCt**	**Gene**	**ΔCt**	**Gene**	**ΔCt**	**Gene**	**ΔCt**	**Gene**	**ΔCt**
*CD49e*	3.2	*CD276*	6.6	*CD117*	10.0	*CD127*	12.5	*CD48*	14.4
*CD120a*	3.4	*CD126*	7.2	*CD123*	10.0	*CD195*	12.5	*CD135*	14.5
*CD44*	3.4	*CD95*	7.2	*CD157*	10.1	*CD197*	12.7	*CD72*	14.5
*CD29*	3.5	*CD36*	7.5	*CD274*	10.2	*CD253*	12.8	*CD278*	14.6
*Sca1*	4.4	*CD49b*	7.8	*CD283*	10.3	*CD53*	12.8	*CD21*	14.7
*CD140a*	4.4	*CD13*	7.9	*CD73*	10.3	*CD103*	13.0	*CD212*	14.7
*CD107a*	5.2	*CD202b*	7.9	*CD80*	10.4	*CD104*	13.1	*CD279*	14.8
*CD140b*	5.2	*CD275*	7.9	*CD133*	10.5	*CD137*	13.1	*CD62L*	14.9
*CD119*	5.3	*CD14*	8.0	*CD180*	10.5	*CD162*	13.3	*CD69*	15.0
*CD147*	5.3	*CD284*	8.2	*CD282*	10.7	*CD210*	13.3	*CD94*	15.0
*CD54*	5.4	*CD49a*	8.3	*CD61*	10.8	*CD265*	13.3	*CD178*	15.1
*CD81*	5.4	*CD90*	8.3	*CD93*	10.9	*CD28*	13.4	*CD193*	15.1
*CD47*	5.6	*CD200*	8.4	*CD115*	11.0	*CD86*	13.4	*CD152*	15.5
*CD106*	6.0	*CD24*	8.4	*CD121b*	11.0	*CD62E*	13.5	*CD23*	15.5
*CD121a*	6.0	*CD71*	8.5	*CD153*	11.5	*CD62P*	13.5	*CD25*	15.6
*CD172a*	6.0	*CD262*	8.5	*CD49f*	11.5	*CD150*	13.9	*CD254*	15.8
*CD266*	6.1	*CD40*	8.9	*CD102*	12.0	*CD22*	13.9	*CD273*	15.9
*CD51*	6.1	*CD107b*	9.0	*CD26*	12.0	*CD122*	14.1	*CD30*	15.9
*CD9*	6.2	*CD34*	9.0	*CD43*	12.2	*CD134*	14.1	*CD38*	16.0
*CD98*	6.3	*CD55*	9.1	*CD88*	12.3	*CD16*	14.2	*CD70*	16.8
*CD105*	6.5	*CD18*	9.2	*CD45*	12.4	*CD244*	14.2	*CD154*	16.9
*CD124*	6.5	*CD184*	9.5	*CD49d*	12.4	*CD252*	14.2	*CD207*	17.0
*CD206*	6.5	*CD223*	9.9	*CD100*	12.5	*CD27*	14.3		

Quantitative PCR was performed using two independently prepared cDNA samples of lin^neg^ cells of bleomycin-induced lungs at day 12. ΔCt, the Ct of a gene of the sample minus the number of Ct of *Actb (actin, beta)* of the sample. Results represent the mean of two experiments.

The expression levels of 13 genes with low Ct value were compared between lin^neg^ cells of bleomycin-injured lungs at day 12 and lin^neg^ cells of saline-treated lungs using QRT-PCR, to identify cell surface markers that could be used to distinguish myofibroblasts from fibroblasts (Table 2). We found that *CD49e*, *CD44*, *CD54*, and *Sca-1* were differentially expressed between these two cell types (fold change > 2 or < 0.5, and *P* < 0.05).

**Table 2:** Identification of cell surface markers distinguishing myofibroblasts and fibroblasts

**Gene**	**ΔCt of lin**^**neg**^**of saline-treated lungs**	**ΔCt of lin**^**neg**^**of bleomycin-injured lungs of day 12**	**ΔΔCt**	**Fold change (Bleo/saline)**
*CD49e*	9.7	6.8	−2.9	5.50^*^
*CD44*	8.4	7.0	−1.4	2.28^*^
*CD29*	7.9	7.1	−0.8	1.60
*CD140b*	8.8	8.8	0.0	1.00
*CD147*	8.8	8.9	0.1	0.94
*CD120a*	6.8	7.0	0.2	0.89
*CD140a*	7.5	8.0	0.5	0.75
*CD107a*	8.2	8.8	0.6	0.70
*CD47*	8.4	9.2	0.8	0.62^*^
*CD81*	8.0	9.0	1.0	0.56^*^
*CD119*	7.8	8.9	1.1	0.52^*^
*CD54*	7.7	9.0	1.3	0.47^*^
*Sca-1*	6.6	8.0	1.4	0.44^*^

ΔCt = Ct of a gene of the sample minus Ct of *18 s rRNA* of the sample. ΔΔCt = ΔCt of lin^neg^ of bleomycin-injured lungs at day 12 minus ΔCt of lin^neg^ of saline-treated lungs. Calculation for fold change in gene levels between bleomycin-injured lungs at day 12 and saline-treated lungs (bleomycin/saline) is described in Methods. QRT-PCR was performed in triplicate using three independently prepared RNA samples. Results represent the mean of three experiments. *, *P* < 0.05.

**Figure 5:**
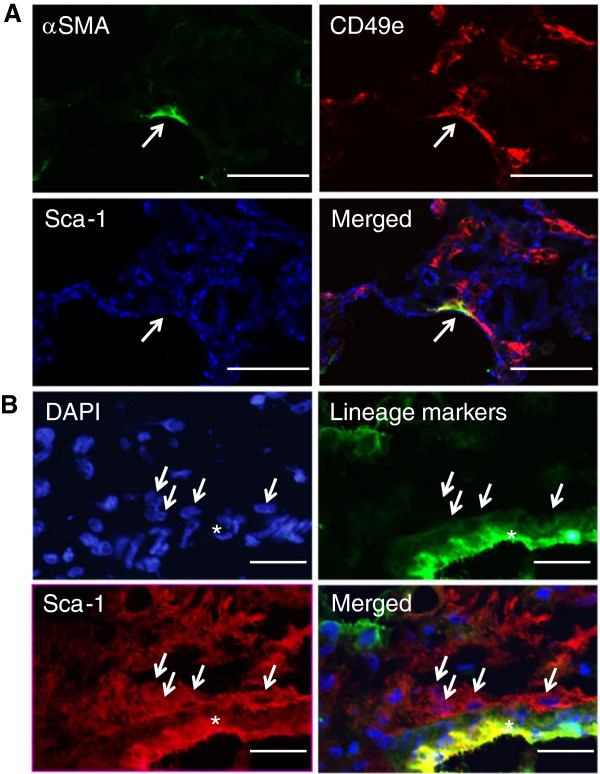
**Exploring cell surface markers of myofibroblasts and fibroblasts. (A)** Snap-frozen bleomycin-injured lungs at day 12 were fixed in acetone and examined by immunofluorescence using anti-α-SMA antibody (upper left), anti-CD49e antibody (upper right), and APC-conjugated anti-Sca-1 antibody (lower left); the three images were merged (lower right). Arrows indicate a myofibroblast. Figures show representative results. Scale bars indicate 100 μm. **(B)** Snap-frozen bleomycin-injured lungs at day 12 were fixed in acetone and examined by immunofluorescence with DAPI (upper left), FITC-conjugated antibodies to lineage-specific cell surface markers (CD31, CD45, EpCAM, TER119, CD146, and Lyve-1) (upper right), and PE-conjugated anti-Sca-1 antibody (lower left); the three images were merged (lower right). Arrows indicate cells that were localized in the adventitia of the pulmonary artery and that were negative for lineage-specific cell surface markers (upper right) but positive for Sca-1 (lower left). Figures show representative results. Scale bars indicate 50 μm. DAPI, 4′,6-diamidino-2-phenylindole.

Immunofluorescence analysis using antibodies against CD49e (fold change: 5.50) and Sca-1 (fold change: 0.44) demonstrated that Sca-1 was expressed in perivascular cells, whereas CD49e, but not Sca-1, was expressed in myofibroblasts in bleomycin-injured lungs (Figures 5A and B). In saline-treated lungs, Sca-1 was expressed in perivascular cells, which were negative for lineage-specific cell surface markers. Our data are consistent with a previous study showing that Sca-1 is expressed in perivascular fibroblasts in normal lung tissue [27].

Because lineage-specific cell surface markers (CD31, CD45, CD146, EpCAM, Lyve-1, and TER119) were not expressed in mesothelial cells, flat epithelial-like cells on the lung surface, we were concerned that lin^neg^ cells might contain many mesothelial cells. However, immunofluorescence analysis using anti-CD49e and anti-Sca-1 antibodies showed that CD49e and Sca-1 were expressed in mesothelial cells at very low levels (Additional file 1: Figure S4), indicating that mesothelial cells can be excluded when anti-CD49e and anti-Sca-1 antibodies are used for the isolation of myofibroblasts and perivascular fibroblasts using FACS.

### Identification of myofibroblast-rich and fibroblast-rich populations

**Figure 6:**
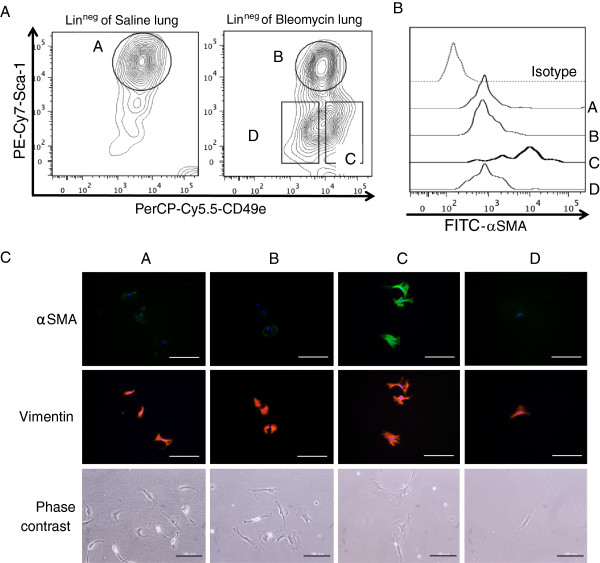
**Identification of myofibroblast-rich and fibroblast-rich populations. (A)** Flow cytometry plots showing the expression of CD49e (*x*-axis) and Sca-1 (*y*-axis) of lin^neg^ cells of saline-treated lungs (left) and bleomycin-injured lungs at day 12 (right). Lin^neg^ Sca-1^high^ of saline-treated lungs (population A) and bleomycin-injured lungs at day 12 (population B) are indicated as circles. Lin^neg^ Sca-1^low^ CD49e^high^ of bleomycin-injured lungs at day 12 (population C) and lin^neg^ Sca-1^low^ CD49e^low^ of bleomycin-injured lungs at day 12 (population D) are indicated as oblongs. Figures show representative results. **(B)** Histogram showing the expression levels of intracellular α-SMA of populations A, B, C, and D using FITC-conjugated anti-α-SMA antibody. As a control, FITC-conjugated isotype control antibody was used. Figures show representative results. **(C)** Sorted cells from populations A, B, C, and D were grown for 36 h, fixed in 4% paraformaldehyde, and subjected to immunocytochemistry using anti-α-SMA antibody (green), anti-vimentin antibody (red), and DAPI (blue). Phase-contrast images of living cells are shown in lower panels. Figures show representative results. Scale bars indicate 100 μm. DAPI, 4′,6-diamidino-2-phenylindole.

Analysis by flow cytometry showed expression levels of CD49e and Sca-1 of lin^neg^ cells of bleomycin-injured lungs at day 12 and saline-treated lungs (Figure 6A). Most lin^neg^ cells of saline-treated lungs expressed Sca-1 at high levels. Conversely, two-thirds of lin^neg^ cells of bleomycin-injured lungs expressed Sca-1 at low levels; the remaining cells expressed Sca-1 at high levels. Lin^neg^ Sca-1^high^ cells (population A) were sorted from saline-treated lungs, and lin^neg^ Sca-1^high^ cells (population B), lin^neg^ Sca-1^low^ CD49e^high^ cells (population C), and lin^neg^ Sca-1^low^ CD49e^low^ cells (population D) were sorted from bleomycin-injured lungs, by FACS. The expression levels of α-SMA of each population were then quantified by flow cytometry. Figure 6B shows that most cells in population C expressed α-SMA at high levels, whereas most cells in the other populations expressed α-SMA at low levels. These results indicated that most cells in population C were myofibroblasts and most cells in populations A, B, and D were not myofibroblasts.

The sorted cells from populations A, B, C, and D were grown on culture plates. On day 1 after sorting, the expression levels of α-SMA and vimentin were analyzed immunocytochemically. As shown in Figure 6C, most adherent cells of populations A, B, and D were α-SMA-negative or showed low expression levels, but were vimentin-positive. In addition, all adherent cells of populations A, B, and D spontaneously differentiated into α-SMA-positive myofibroblast-like cells after 7 days of culture in the presence or absence of TGF-β (data not shown) as previously reported [28], suggesting that adherent cells of populations A, B, and D were originally fibroblasts. Most adherent cells of population C were α-SMA-positive (Figure 6C), demonstrating that they were myofibroblasts. We did not detect any CD31-positive endothelial cells, CD146-positive pericytes, EpCAM-positive epithelial cells, calretinin-positive mesothelial cells, CD45-positive macrophages, or dendritic cells among the adherent cells of populations A, B, C, and D by immunocytochemical analysis (data not shown).

These findings indicated that fibroblasts were highly enriched in populations A and B and that myofibroblasts were highly enriched in population C. Because the number of adherent cells of population D was much smaller than that of the other populations, we did not investigate them further.

### *In-vitro* and *in-vivo* proliferative capacity of myofibroblasts and fibroblasts

**Figure 7:**
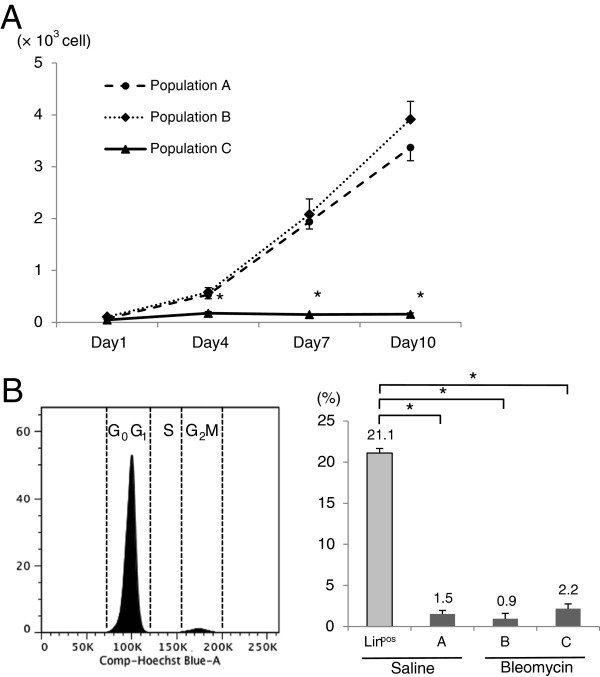
***In-vitro *and *in-vivo *proliferative capacity of myofibroblasts and fibroblasts.(A)** Two hundred sorted cells from populations A, B, and C were grown in 12-well plates. The graph represents the number of adherent cells of each population, which were counted on days 1, 4, 7, and 10 after sorting. Experiments were performed in triplicate. Results represent the mean (± s.d.) of three experiments. *, *P* < 0.01. **(B)** The left figure shows the cell-cycle distribution of the DNA histogram from Hoechst 33342-stained cells of population A. The high peak on the left side represents cells in the G_0_G_1_ phase, and the low peak on the right side represents cells in the G_2_M phase. The region between the two peaks represents cells in the S phase. The proportion of cells in the G_2_M phase was compared among lineage-specific cell surface marker-positive cells of saline-treated lungs (lineage-positive cells of saline-treated lungs), and populations A, B, and C (right). The bar graph represents the proportion of cells in the G_2_M phase. Experiments were performed in triplicate. Results represent the mean (± s.d.) of three experiments. Asterisks indicate *P* < 0.001. s.d., standard deviation.

To examine their *in-vitro* proliferative capacity, the adherent cells of each population were counted on days 1, 4, 7, and 10 after FACS (Figure 7A). Interestingly, fibroblasts of saline-treated lungs and bleomycin-injured lungs at day 12 proliferated, whereas myofibroblasts of bleomycin-injured lungs at day 12 did not proliferate *in vitro*.

To assess the *in-vivo* cell-cycle status, we performed a quantitative cell-cycle assay using Hoechst 33342 (Figure 7B). The proportion of cells in the G_2_M phase of populations A, B, and C was similar (1.5%, 0.9%, and 2.2%, respectively), but was markedly lower than that of lineage-specific cell surface marker-positive cells in saline-treated lungs (Figure 7B) and bleomycin-injured lungs (data not shown).

Therefore, we concluded that fibroblasts and myofibroblasts had low proliferative capacity *in vivo*, and that fibroblasts of bleomycin-injured and saline-treated lungs proliferated *in vitro*, but myofibroblasts of bleomycin-injured lungs did not proliferate *in vitro*.

### Expression profiling of genes for collagen, collagen synthesis enzymes, and chemokines of myofibroblasts and fibroblasts

The expression of genes for collagen and collagen synthesis enzymes of directly isolated myofibroblasts and fibroblasts was investigated. The pathway of the posttranslational modification of collagen is described elsewhere [29,30]. In short, after having been catalyzed by intracellular enzymes, that is, prolyl 4-hydroxylase (P4h), procollagen lysyl hydroxylase (Plod), and heat shock protein 47, procollagen produced by fibroblasts and myofibroblasts is catalyzed by a procollagen proteinase (Adamts 2) and lysyl oxidase (Lox) or members of the Lox-like (Loxl) family, after which collagen fibrils are formed.

**Figure 8:**
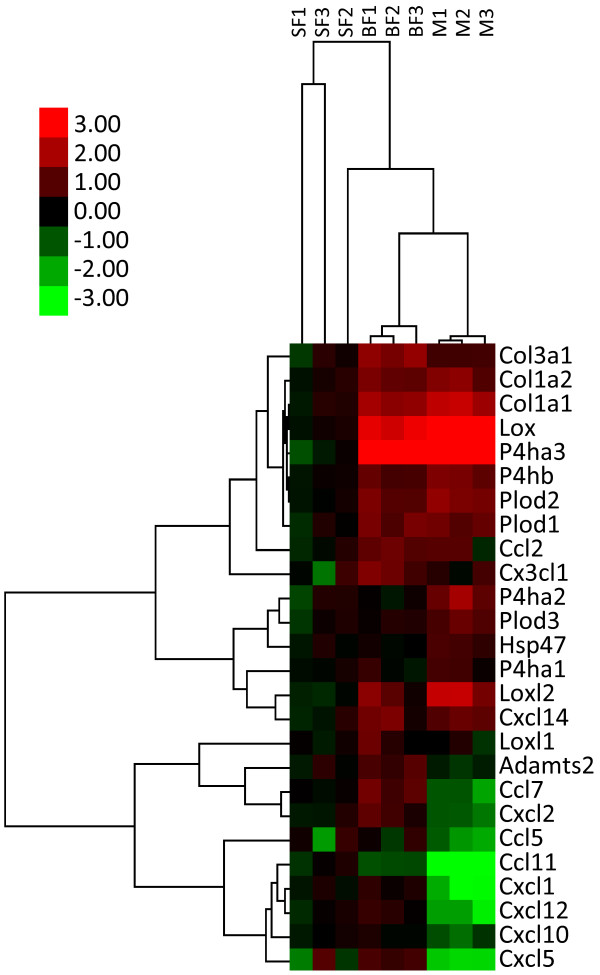
**Expression profiling of genes for collagen, collagen synthesis enzymes, and chemokines of directly isolated cells.** Cluster and dendrogram analyses were performed among three types of directly isolated cells, that is, fibroblasts of saline-treated lungs (SF1, SF2, and SF3), fibroblasts of bleomycin-injured lungs at day 12 (BF1, BF2, and BF3), and myofibroblasts of bleomycin-injured lungs at day 12 (M1, M2, and M3). QRT-PCR was performed in triplicate using three independently prepared RNA samples. The original raw data are shown in Additional file 1: Tables S4 and S5.

By using qRT-PCR, we compared the expression levels of genes for collagen (*Col1a1*, *Col1a2*, and *Col1a3*) and collagen synthesis enzymes (*P4ha1*, *P4ha2*, *P4ha3*, *P4hb*, *Plod1*, *Plod2*, *Plod3*, *heat shock protein 47*, *Adamts2*, *Lox*, *Loxl1*, and *Loxl2*) among directly isolated fibroblasts of saline-treated lungs, fibroblasts of bleomycin-injured lungs, and myofibroblasts of bleomycin-injured lungs (Additional file 1: Table S3). As shown in Figure 8 and Table 3, the expression levels of *Col1a1*, *Col1a2*, *P4ha3*, *Plod1*, *Plod2*, *Lox*, and *Loxl2* were significantly higher (fold change > 2.0, *P* < 0.05) in fibroblasts and myofibroblasts of bleomycin-injured lungs than in fibroblasts of saline-treated lungs. However, the expression pattern of genes for collagen and collagen synthesis enzymes of myofibroblasts was similar to that of fibroblasts of bleomycin-injured lungs. These findings indicated that the ability of fibroblasts and myofibroblasts of bleomycin-injured lungs to produce type 1 collagen might be similar, but was greater than that of fibroblasts of saline-treated lungs.

**Table 3:** Differentially expressed genes among three types of directly isolated cell

**B fibroblasts/S fibroblasts**		**Myofibroblasts/S fibroblasts**		**Myofibroblasts/B fibroblasts**	
**Gene**	**Fold change**	**Gene**	**Fold change**	**Gene**	**Fold change**
*Col1a1*	2.97	*Col1a1*	3.91	*P4ha2*	2.68
*Col1a2*	2.05	*Col1a2*	2.28	*Adamts2*	0.42
*Col3a1*	3.02	*P4ha2*	2.68	*Ccl5*	0.32
*P4ha3*	11.76	*P4ha3*	21.01	*Ccl7*	0.19
*Plod1*	2.43	*P4hb*	2.49	*Ccl11*	0.02
*Plod2*	2.23	*Plod1*	2.30	*Cxcl1*	0.12
*Lox*	5.90	*Plod2*	2.88	*Cxcl2*	0.27
*Loxl2*	2.69	*Plod3*	2.06	*Cxcl5*	0.11
*Ccl2*	2.27	*Lox*	8.08	*Cxcl10*	0.49
*Ccl7*	2.10	*Loxl2*	5.27	*Cxcl12*	0.17
		*Ccl7*	0.40		
		*Ccl11*	0.01		
		*Cxcl1*	0.16		
		*Cxcl2*	0.46		
		*Cxcl12*	0.22		
		*Cxcl14*	2.22		

Differentially expressed genes in fibroblasts of bleomycin-injured lungs at day 12 (B fibroblasts) relative to fibroblasts of saline-treated lungs (S fibroblasts), myofibroblasts of bleomycin-injured lungs at day 12 relative to S fibroblasts, and myofibroblasts relative to B fibroblasts by a factor of > 2.0 or < 0.5 and *P* < 0.05. QRT-PCR was performed in triplicate using three independently prepared RNA samples. Results represent the mean of three experiments.

Many cell types, including fibroblasts, secrete various chemokines to recruit inflammatory cells to injured lesions [31]. To our knowledge, at least 11 chemokines (*Ccl2*, *Ccl5*, *Ccl7*, *Ccl11*, *Cxcl1*, *Cxcl2*, *Cxcl5*, *Cxcl10*, *Cxcl12*, *Cxcl14*, and *Cx3cl1*) (Additional file 1: Table S3) are secreted by lung fibroblasts *in vivo* or *in vitro*, as shown in previous studies [32-40]. Figure 8 and Table 3 show the expression levels of 11 chemokine genes of the three types of directly isolated cell. Interestingly, the expression levels of eight chemokine genes of myofibroblasts were decreased when compared with those of fibroblasts of bleomycin-injured lungs.

Taken together, the qRT-PCR results suggest that fibroblasts of saline-treated lungs, fibroblasts of bleomycin-injured lungs, and myofibroblasts are functionally different.

### Expression profiling of genes of directly isolated myofibroblasts and cultured fibroblasts

**Figure 9:**
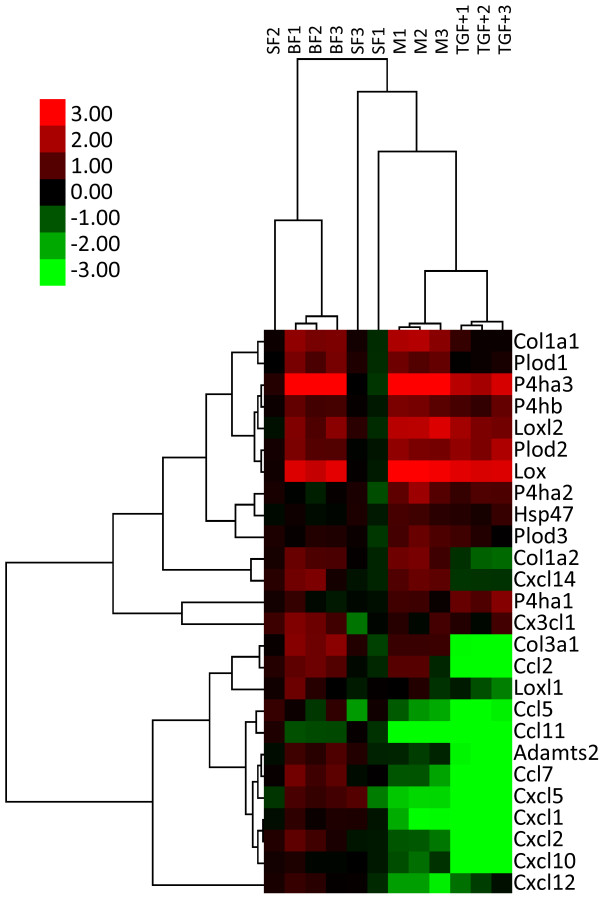
**Expression profiling of directly isolated myofibroblasts and cultured fibroblasts.** Cluster and dendrogram analyses were performed among three types of directly isolated cells (SF1, SF2, SF3, BF1, BF2, BF3, M1, M2 and M3) and myofibroblasts cultured in the presence of TGF-β1 (TGF + 1, TGF + 2, TGF + 3). QRT-PCR was performed in triplicate using three independently prepared RNA samples. The original raw data are shown in Additional file 1: Tables S4 and S5.

After isolated fibroblasts of saline-treated lungs had been cultured for 7 days, the cells differentiated into α-SMA-positive myofibroblast-like cells in the presence of TGF-β1. Because it is unknown whether the gene expression pattern of cultured cells is altered by the effect of culture conditions, we compared the gene expression patterns of the three types of directly isolated cells with that of fibroblasts of saline-treated lungs that had been cultured in the presence of TGF-β1. As expected, among the three types of cell, the gene expression pattern of cultured myofibroblast-like cells was most similar to that of directly isolated myofibroblasts but many genes were differentially expressed between the cell types (Figure 9 and Table 4).

**Table 4:** Differentially expressed genes between cultured fibroblasts and directly isolated myofibroblasts

**Gene**	**Fold change**
*Col1a1*	0.32
*Col1a2*	0.23
*Col3a1*	0.06
*P4ha3*	0.23
*Adamts2*	0.16
*Ccl2*	0.03
*Ccl5*	0.23
*Ccl7*	0.09
*Ccl11*	0.002
*Cxcl1*	0.01
*Cxcl2*	0.01
*Cxcl5*	0.01
*Cxcl10*	0.01
*Cxcl14*	0.31

Differentially expressed genes in cultured fibroblasts (α-SMA-positive myofibroblast-like cells) relative to directly isolated myofibroblasts by a factor of > 2.0 or < 0.5 and *P* < 0.05. QRT-PCR was performed in triplicate using three independently prepared RNA samples. Results represent the mean of three experiments.

The expression patterns of collagen genes (*Col1a1*, *Col1a2*, and *Col3a1*) and most chemokine genes were different, indicating that *in-vitro* culture conditions affected gene expression.

## Discussion

On the basis of the information obtained from qRT-PCR, immunofluorescence, flow cytometry, and cell culture experiments, we demonstrated that fibroblasts and myofibroblasts of saline-treated lungs and bleomycin-injured lungs in the proliferative phase were enriched in the lin^neg^ Sca-1^high^ population and in the lin^neg^ Sca-1^low^ CD49e^high^ population, respectively. Approximately 46,000 ± 17000 cells (*N* = 3) cells of a fibroblast-rich population can be isolated from a lung of a saline-treated mouse and approximately 34,000 ± 15000cells (*N* = 3) cells of a fibroblast-rich population and 16,000 ± 5000cells (*N* = 3) cells of a myofibroblast-rich population can be isolated from a lung of a bleomycin-injured mouse using this isolation method. Because *in-vitro* culture conditions affected gene expression (Figure 9), it is reasonable to postulate that the global gene expression signature of directly isolated cells by FACS reflects the *in-vivo* transcriptional state more closely than that of cultured cells.

Bleomycin-injured lungs of mice differ from human idiopathic pulmonary fibrosis (IPF) lungs in their histological and pathological features [41,42]. For example, fibroblastic foci, which are histological hallmarks of IPF, are rarely found in bleomycin-injured lungs. Fibroblastic foci consist of myofibroblasts in myxomatous stroma and are used to distinguish IPF from other interstitial pneumonias. Immunohistochemical analysis demonstrated that CD49e was expressed in myofibroblasts in most fibroblastic foci of human IPF, as shown in a previous study (Additional file 1: Figure S5D) [43]. However, CD49e was also expressed in epithelial cells overlaying the fibroblastic foci and endothelial cells. Therefore, an appropriate combination of cell surface markers should be required for isolating myofibroblasts in fibroblastic foci of IPF (Additional file 1: Figure S5E).

In addition, an immunohistochemical analysis of a human IPF lung demonstrated that CD49e was not expressed in myofibroblasts within a fibrotic scar (Additional file 1: Figure S5I), indicating that myofibroblasts might be divided into two types in terms of the expression of CD49e, that is, CD49e-positive myofibroblasts found in fibroblastic foci and CD49e-negative myofibroblasts found within a fibrotic scar. CD49e-positive and CD49-negative myofibroblasts might represent young myofibroblasts and mature myofibroblasts, respectively.

The bleomycin-injured lung model used in this study has a limitation with regard to isolating myofibroblasts at the remodeling phase because we failed to obtain bleomycin-treated mice around days 21 and 28 after treatment (Figure 1A). Typically, extensive fibrosis occurs in bleomycin-injured lungs between 21 and 28 days after intratracheal administration of bleomycin, as shown in previous studies [21,22]. Therefore, the sex, age and body mass of the mice used and the amount of bleomycin used for intratracheal administration should be considered to obtain bleomycin-treated mice at the remodeling phase [44].

It would be of interest to compare the biological characteristics and gene signature of young myofibroblasts at the proliferative phase with those of mature myofibroblasts at the remodeling phase in bleomycin-injured lungs. Similarly, the biological properties and gene signature of CD49e-positive myofibroblasts of fibroblastic foci and CD49e-negative myofibroblasts within fibrotic scars would improve our understanding of the biology and pathology of myofibroblasts in IPF lungs, in addition to other fibrotic diseases.

*In-vivo* cell-cycle analysis using Hoechst 33342 showed that the proliferative capacity of myofibroblasts of bleomycin-injured lungs of day 12 was low and comparable with that of fibroblasts (Figure 7B). However, only myofibroblasts did not have proliferative capacity *in vitro*, as shown in Figure 7A. The difference of *in-vitro* proliferative capacity between fibroblasts and myofibroblasts leads us to assume that the optimal culture conditions for the proliferation of myofibroblasts are different from those of fibroblasts, including growth factors and oxygen levels in culture medium, and extracellular matrix with which the surface of a culture plate is coated.

Although the expression levels of most genes for collagen and collagen synthesis enzymes were similar between myofibroblasts and fibroblasts of bleomycin-injured lungs at day 12 (Figure 8), some genes were differentially expressed. The expression levels of *P4ha2* and *P4ha3* of myofibroblasts were significantly higher than those of fibroblasts of bleomycin-injured lungs (*P4ha2*; fold change = 2.68, *P* < 0.05, *P4ha3*; fold change = 1.79, *P* < 0.01) (Additional file 1: Table S4). P4h is composed of two identical α (isoenzymes P4ha1, P4ha2, and P4ha3) and two β (P4hb) subunits and is essential for the stability of the triple helix of collagen. Because P4h is the rate-limiting enzyme of the pathway of posttranslational modification of collagen, increased expressions of *P4ha2* and *P4ha3* in myofibroblasts might result in increased production of collagen.

We found that the expression levels of *P4ha3* were markedly increased in fibroblasts and myofibroblasts of bleomycin-injured lungs (Table 3). However, immunohistochemical analysis demonstrated that the P4ha3 expression in bleomycin-injured lungs was markedly increased in most cells, whereas its expression in saline-treated lungs was low (Additional file 1: Figure S6), indicating that the increased expression of P4ha3 was not specific to fibroblasts and myofibroblasts of bleomycin-injured lungs.

The expression levels of *Lox* and *Loxl2* were much higher in myofibroblasts and fibroblasts of bleomycin-injured lungs than in fibroblasts of saline-treated lungs (Table 3), indicating that collagen produced by fibroblasts and myofibroblasts of bleomycin-injured lungs might be easily cross-linked because of the increased levels of Lox and Loxl2. A recent study showed that lung fibrosis of mice induced by bleomycin, and liver fibrosis, was inhibited by administration of anti-Loxl2 monoclonal antibody [45]. In addition, previous studies of the effect of β-aminopropionitrile, an inhibitor of Lox, in lung fibrosis models of rat and hamster have demonstrated that bleomycin-induced fibrosis was attenuated by administration of β-aminopropionitrile [46,47]. Thus, Lox and Loxl2 might be useful targets for the development of new antifibrotic drugs [48].

The origin of myofibroblasts remains controversial [49]. Myofibroblasts have been thought to be a heterogeneous population [7] composed of resident fibroblasts in the perivascular area [50], epithelial cells (by epithelial-mesenchymal transition) [51], endothelial cells (by endothelial-mesenchymal transition) [52], mesothelial cells [53], and circulating fibrocytes derived from bone marrow [54,55]. In this study, the heterogeneity of myofibroblasts was not taken into account. Further investigations are needed to clarify the function of each subpopulation of myofibroblasts.

**Figure 10:**
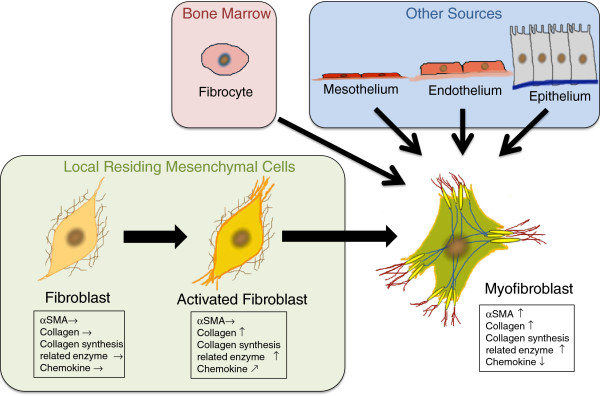
**Model of myofibroblast development.** Resident fibroblasts are activated when the tissue is injured, and then begin to produce more collagen and chemokines. After activated fibroblasts differentiate into myofibroblasts, they still produce collagen, but they do not produce chemokines, as do fibroblasts and activated fibroblasts. Other types of cell, such as mesothelial cells, endothelial cells, epithelial cells, and circulating fibrocytes also participate in myofibroblast development.

Here, we demonstrated functional similarities and differences among directly isolated fibroblasts of saline-treated lungs, fibroblasts of bleomycin-injured lungs, and myofibroblasts of bleomycin-injured lungs (Figure 10). Resident fibroblasts are activated on tissue injury and start to express more genes for collagen and collagen synthesis enzymes. After activated fibroblasts differentiate into myofibroblasts, they continue to express genes for collagen and collagen synthesis enzymes to the similar extent as activated fibroblasts. Myofibroblasts have a decreased ability to express chemokine genes; however, the ability of collagen production is conserved after differentiation.

## Conclusions

In this study, we report a direct method for isolating myofibroblasts and fibroblasts from bleomycin-injured lungs in mice at day 12 after injury. We demonstrated differences in the proliferative capacity between fibroblasts and myofibroblasts directly isolated from fibrotic lungs. Analysis of the expression levels of genes for collagen, collagen synthesis enzymes, and chemokines indicated functional similarities and differences between myofibroblasts and fibroblasts. The direct isolation method provides useful information for a better understanding of the biological properties of myofibroblasts and fibroblasts of injured tissues.

## Methods

### Animal experiment

This study was approved by the Animal Care and Use Committee of Hamamatsu University School of Medicine. All animal experiments were performed in accordance with the guidelines for animal experiments of Hamamatsu University School of Medicine. All efforts were made to minimize the number of animals used and any discomfort.

C57BL/6 mice were purchased from Japan SLC (Shizuoka, Japan), and NG2DsRedBAC transgenic mice were purchased from Jackson Laboratory (Bar Harbor, ME). The mice were maintained in a pathogen-free mouse facility. Female C57BL/6 mice of 10 to 12 weeks of age (20 to 25 g body mass) were intraperitoneally anesthetized using pentobarbital sodium (Kyoritsu Seiyaku Co, Tokyo, Japan) (77.8 μg/g body mass), followed by a single intratracheal injection of 2 mg/kg of bleomycin sulfate (Wako, Osaka, Japan) in 50 μl of sterile phosphate-buffered saline [56]. The body mass of mice treated with bleomycin decreased after administration of bleomycin. Mice were killed by cervical dislocation on day 12 after bleomycin or saline treatment.

### Hydroxyproline assay

Changes of lung collagen were determined by analysis of hydroxyproline according to the manufacturer’s instructions with modifications (BioVision, Milpitas, CA). Lungs were harvested on day 12 after bleomycin or saline treatment and homogenized in distilled water, using 100 μl for every 10 mg of tissue. 160 μl 7.5 N HCl was added to 40 μl sample homogenate, and hydrolyzed in a pressure-tight vial at 120°C for 3 hours in a dry heat oven. We then followed the manufacturer’s instructions.

### Bronchoalveolar lavage (BAL) fluid

After mice were killed, the trachea was cannulated using a 20-gage catheter. BAL was performed twice with 1.0 ml of ice-cold PBS with an 80% recovery rate. The BAL fluid was centrifuged, and supernatant was collected. Total cells were counted on a hemocytometer in the presence of 0.4% trypan blue (Sigma-Aldrich, St Louis, MO). For differential cell counting, cells were smeared on glass slides, fixed, and stained with Giemsa solution. The number of macrophages, neutrophils and lymphocytes in 400 cells was counted based on morphology.

### Analysis of cytokines in BAL fluid

The concentrations of IL-6 and TGFβ1 in BAL fluid were determined by ELISA (R&D systems, Minneapolis, MN).

### Antibodies

The primary antibodies used in this study were purchased from eBioscience (San Diego, CA), Biolegend (San Diego, CA), Miltenyi Biotechnology (Bergisch Gladbach, Germany), Abcam (Cambridge, UK), Sigma-Aldrich (St Louis, MO), Santa Cruz Biotechnology (Dallas, TX), Millipore (Billerica, MA), Cell Signaling Technology (Danvers, MA), DakoCytomation (Carpinteria, CA), Molecular Probes (Grand Island, NY), and Epitomics (Burlingame, CA). Detailed information on the antibodies is shown in Additional file 1: Table S1.

### Immunofluorescence

After lungs of C57BL/6 mice in optimal cutting temperature compound (Sakura Finetek, Tokyo, Japan) had been frozen at −80°C in organic solvent, 6 μm thick slices, sectioned at −20°C using a cryostat, were fixed in cold acetone for 10 min and dried for 20 min at room temperature. After lungs of NG2DsRedBAC transgenic mice had been fixed in 10% buffered formalin overnight, 6 μm thick slices sectioned at −20°C, using a cryostat, were dried for 20 min at room temperature. The sections were incubated in blocking solution (10% goat serum in PBS) for 30 min at room temperature, and then incubated with primary antibody and 1 μg/ml 4′,6-diamidino-2-phenylindole (DAPI) (Sigma-Aldrich) in blocking solution for 30 min at room temperature, and then washed in PBS. The sections were incubated with unconjugated primary antibody followed by incubation with Alexa-fluor conjugated secondary antibody. The sections were mounted in Prolong Gold (Molecular Probes) and imaged with Olympus BX51 fluorescence microscope (Olympus, Tokyo, Japan). Images were captured with a DP71 CCD camera (Olympus), processed with DP control and DP manager software (Olympus), and then postprocessed with Adobe Photoshop CS3 (Adobe Systems, Inc., San Jose, CA).

### Immunohistochemistry

Lungs were fixed in 10% buffered formalin, embedded in paraffin, and sectioned to 4 μm thick slices. After the sections were deparaffinized, they were initially incubated in 3% hydrogen peroxide solution for 20 min at room temperature. Antigen retrieval was performed according to the manufacturer’s instructions. The sections were incubated with primary antibody in blocking solution (10% goat serum and 0.1% Triton X-100 in PBS) for 30 min at room temperature. After washing in PBS, the sections were incubated with peroxidase-conjugated universal immuno-enzyme polymer, anti-mouse or anti-rabbit solution (Nichirei Biosciences), and then visualized by 3,3-diaminobenzidine (Sigma-Aldrich), and counter-stained with hematoxylin.

### Fluorescence activating cell sorting (FACS)

Fat tissue, bronchus, pulmonary veins, and arteries were removed from lungs. The lungs were incubated with 200 U/ml collagenase type 2 (Worthington, Lakewood, NJ) and 100 U/ml DNase 1 (Worthington) for 30 min at 37°C in Dulbecco’s PBS (Gibco, Carlsbad, CA) and cut using a gentleMACS^TM^ Dissociator (Miltenyi Biotechnology) according to the manufacturer’s instructions. After cells were filtered through a nylon screen (BD Bioscience, San Diego, CA) to remove cell aggregates of, cells were centrifuged and rinsed twice by FACS buffer (1% HEPES buffer, 2% heat-inactivated FCS, 120 μg/ml penicillin, 100 μg/ml streptomycin in HBSS). Cells were incubated with biotin-conjugated anti-CD49e antibody (×150), PE-Cy7-conjugated anti-Sca1 (×600) antibody, and antibodies against lineage-specific cell surface markers, that is, FITC-conjugated anti-CD146 antibody (×10), FITC-conjugated anti-Lyve-1 antibody (×100), APC-conjugated anti-EpCAM antibody (×100), APC-conjugated anti-CD31 antibody (×100), APC-conjugated anti-CD45 antibody (×100), and APC-conjugated anti-TER119 antibody (×100) for 30 min on ice. After centrifuging and rinsing twice in FACS buffer, the cells were incubated with PerCP-Cy5.5-conjugated streptavidin (×300) for 30 min on ice. After centrifuging and rinsing twice by FACS buffer, all sorting and analysis were performed on a FACSAria (BD Bioscience). To obtain higher purity, the sorted sample was subjected to a second round of sorting. The isotype antibodies were used as a control experiment.

To analyze cell-cycle status using Hoechst 33342, cells were incubated with 1 μg/ml Hoechst 33342 (Sigma-Aldrich) and 50 μg/ml verapamil (Sigma-Aldrich) to block MDR-mediated Hoechst efflux [57]. Cells were incubated for 45 min at 37°C and agitated every 5 min to prevent settling. After incubation, the cells were put on ice and incubated with antibodies as described above. Hoechst staining of cells was assayed by flow cytometry. The formula for calculation of the proportion of cells in the G_2_M phase is:

$$G_{2}\mathrm{M\%}=\left(\mathrm{Number}\;\mathrm{of}\;\mathrm{cells}\;\mathrm{in}\;\mathrm{the}\;G_{2}M\;\mathrm{phase}\;/\;\mathrm{Number}\;\mathrm{of}\;\mathrm{cells}\;\mathrm{in}\;\mathrm{the}\;G_{0}G_{1},\;S,\;\mathrm{and}\;G_{2}M\;\mathrm{phases}\;\mathrm{combined}\right)\;\times \;100$$

### Quantitative RT-PCR

Total RNA was extracted from 5,000 freshly isolated cells using Trizol (Invitrogen) with glycogen as a carrier. RNA was extracted following the manufacturer’s instructions. The extracted RNA was treated for 20 min at 37°C with RNase free DNase 1 (Ambion, Austin, TX) in the presence of RNase inhibitor (Invitrogen, Carlsbad, CA). The RNA was purified with RNeasy Mini Kit (Qiagen, Hilden, Germany) according to the manufacturer’s instructions. After first-strand cDNA had been synthesized by SuperScript Reverse Transcriptase (Invitrogen) with random primers (Invitrogen), cDNA equivalent to 100 cells was used for each PCR reaction. Gene-specific primers were designed by Primer 3 (v.0.4.0) software [58] to generate short amplicons (100 to 150 bps). The PCR reactions were performed using a SYBR Green qRT-PCR Kit (Applied Biosystems, Foster City, CA). The sequences of the gene-specific primers are shown in Additional file 1: Tables S2 and S3.

The PCR cycling program consisted of one cycle of 95°C for 10 min, 40 cycles of 95°C for 15 s and 60°C for one min, and one cycle of 95°C for 15 s and 60°C for one min (ABI StepOnePlus). The cycle threshold (Ct) for each gene was measured. cDNA equivalent to ten cells was used to measure Ct of *18 s ribosomal RNA* (*rRNA*). The QRT-PCR products were separated in 2% agarose gels to confirm the presence of a single band of the expected size. QRT-PCR was performed in triplicate using three independent RNA samples. However, qRT-PCR for 114 genes (Table 1 and Additional file 1: Table S2) was performed in duplicated using two independent RNA samples.

To estimate the relative difference in gene expression between samples, we assumed that the efficiency of amplification was 90%, a typical value [59,60]. The formula for fold change in a gene levels between samples is:

Fold change = 1.8^*x*^

where:

$$x=\left(\mathrm{Ct}\;\mathrm{of}\;a\;\mathrm{gene}\;\mathrm{of}\;\mathrm{sample}\;A\;-\;\mathrm{Ct}\;\mathrm{of}\;18s\;\mathrm{rRNA}\;\mathrm{of}\;\mathrm{sample}\;A\right)-\left(\mathrm{Ct}\;\mathrm{of}\;a\;\mathrm{gene}\;\mathrm{of}\;\mathrm{sample}\;B\;-\;\mathrm{Ct}\;\mathrm{of}\;18s\;\mathrm{rRNA}\;\mathrm{of}\;\mathrm{sample}\;B\right)$$

Hierarchical clustering analysis was conducted on the log_2_-transformed fold change values of qRT-PCR using Cluster 3.0 software [61]. The results of clustering analysis were presented in the form of a dendrogram using Java TreeView software [62,63].

### Quantification of intracellular proteins

Isolated 10,000 lin^neg^ cells were centrifuged and fixed in 10% buffered formalin for 15 min at room temperature, and then permeabilized for 5 min using IntraPrep (Beckman Coulter, Brea, CA), according to the manufacturer’s instructions. After centrifuging and rinsing in FACS buffer, the cells were incubated with FITC-conjugated anti-α-SMA antibody for 30 min at room temperature. After centrifuging and rinsing in FACS buffer, FITC fluorescence intensity of cells was measured using the FACSAria. Cells were incubated with unconjugated anti-vimentin antibody (rabbit monoclonal) followed by incubation with Alexa-fluor 488-conjugated anti-rabbit antibody for 30 min at room temperature. Flow cytometry analysis was performed in triplicate using three independent samples.

### Cell culture and immunocytochemistry

Isolated batches of 200 cells were grown in DMEM (Gibco) supplemented with 1× Glutamax (Gibco), 120 μg/ml penicillin, 100 μg/ml streptomycin, and 20% heat-inactivated FCS (Gibco) on 12-well culture plates (CELLSTAR, Greiner bio-one, Kremsmünster, Austria) at 37°C under 5% CO_2_ in the absence or presence of TGF-β1 (10 ng/ml) (R&D Systems). On days 1, 4, 7 and 10 after sorting, adherent cells were fixed in 4% paraformaldehyde for 10 min at room temperature and stained with 1.0 μg/ml DAPI. Cell counting was performed in triplicate using three independent samples.

For immunocytochemistry, isolated batches of 8,000 cells were grown for 36 hours in a 24-well plate, and adherent cells were fixed in 4% paraformaldehyde for 10 min at room temperature, permeabilized with 0.5% Triton X-100 in PBS for 5 min at room temperature, and incubated in blocking solution (10% goat serum and 0.1% Triton X-100 in PBS) for 30 min at room temperature, and then incubated with unconjugated anti-α-SMA antibody and unconjugated anti-vimentin antibody followed by incubation with Alexa-fluor 488-conjugated anti-mouse IgG_2a_ antibody and Alexa-fluor 546-conjugated anti-rabbit antibody. Cells were imaged with an Olympus IX71 fluorescence microscope (Olympus). Images were captured with a DP70 camera (Olympus), and then postprocessed with Adobe Photoshop CS3.

### Statistical analysis

The data shown represent the means ± standard deviation (s.d.) of at least three independent experiments. Statistical analysis is performed using an unpaired Student’s *t* test (two-tailed). *P* < 0.05 is considered statistically significant.

## Abbreviations

α-SMA: α-smooth muscle actin; BAC: bacterial artificial chromosome; BAL: bronchoalveolar lavage; Col1A1: type 1 collagen A1; Ct: cycle threshold; DAPI: 4′,6-diamidino-2-phenylindole; DMEM: Dulbecco’s modified Eagle medium; ELISA: enzyme-linked immunosorbent assay; EpCAM: epithelial cell adhesion molecule; FACS: fluorescence-activated cell sorting; : fetal calf serum; H & E: hematoxylin and eosin; HBSS: Hanks’ balanced salt solution; IL-6: interleukin 6; IPF: idiopathic pulmonary fibrosis; lin: lineage-specific cell surface markers; Lox: lysyl oxidase; Loxl: Lox-like; Lyve-1: lymphatic vessel endothelial hyaluronan receptor; NG2: neuron-glial antigen 2; P4h: prolyl 4-hydroxylase; PBS: phosphate-buffered saline; Plod: procollagen lysyl hydroxylase; qRT-PCR: quantitative reverse transcription polymerase chain reaction; TGF-β: transforming growth factor β.

## Competing interests

All authors declare that they have no competing interests.

## Authors’ contributions

TA, TS, KC, and TI conceived and designed the study. TA, YA, HK, IK, SM, MS, KS, and TI acquired the data acquisition. TA, YA, TS, KC, and TI analyzed and interpreted the data. TA, TS, KC, and TI wrote and reviewed the manuscript. All authors read and approved the final manuscript.

## Supplementary Material

Additional file 1 **Figure S1.** CD146 expression in lung. **Figure S2**. CD146 is a lineage-specific cell surface marker of NG2-positive pericytes and vascular smooth muscle cells in lung. **Figure S3**. FACS gating strategy. **Figure S4**. CD49e and Sca-1 are not expressed in mesothelial cells. **Figure S5**. Expression levels of α-SMA, Col1A1, CD49e, and lineage-specific cell surface markers in myofibroblasts in lung with IPF. **Figure S6**. Increased expression levels of P4ha3 in many cell types in bleomycin-injured lungs. **Table S1**. Antibodies used. **Table S2**. Details of 114 genes for cell surface markers. **Table S3**. Details of genes for collagen, collagen synthesis enzymes, and chemokines. **Table S4**. Raw qRT-PCR data of genes for collagen and collagen synthesis enzymes of the different cell types. **Table S5**. Raw qRT-PCR data of chemokine genes of the different cell types. Click here for file
