# Supplementary material for: Direct isolation of myofibroblasts and fibroblasts from bleomycin-injured lungs reveals their functional similarities and differences
Source: Fibrogenesis Tissue Repair. 2013 Aug 8;6:15. doi: 10.1186/1755-1536-6-15 (PMC3751789; doi:10.1186/1755-1536-6-15)
Supplement: Additional file 1 — Figure S1. CD146 expression in lung. Figure S2. CD146 is a lineage-specific cell surface marker of NG2-positive pericytes and vascular smooth muscle cells in lung. Figure S3. FACS gating strategy. Figure S4. CD49e and Sca-1 are not expressed in mesothelial cells. Figure S5. Expression levels of α-SMA, Col1A1, CD49e, and lineage-specific cell surface markers in myofibroblasts in lung with IPF. Figure S6. Increased expression levels of P4ha3 in many cell types in bleomycin-injured lungs. Table S1. Antibodies used. Table S2. Details of 114 genes for cell surface markers. Table S3. Details of genes for collagen, collagen synthesis enzymes, and chemokines. Table S4. Raw qRT-PCR data of genes for collagen and collagen synthesis enzymes of the different cell types. Table S5. Raw qRT-PCR data of chemokine genes of the different cell types. [file 1755-1536-6-15-S1.docx]

**Additional files**

**Additional file 1: Figure S1**

**CD146 is expressed in pericytes, vascular smooth muscle cells, and bronchial smooth muscle cells**

**(A)** Formalin-fixed normal lungs of NG2DsRedBAC transgenic mice, which express a red fluorescent protein (DsRed) under the control of the mouse NG2 promoter/enhancer on a bacterial artificial chromosome (BAC), are shown (left) and were examined by immunofluorescence with anti-α-SMA antibody (middle), and both images were merged into 1 image with a DAPI-stained image (blue, right). α-SMA was not expressed in NG2-positive pericytes of lung capillaries, whereas NG2 and α-SMA were expressed in vascular smooth muscle cells of the pulmonary artery (asterisk).

**(B)** Snap-frozen saline-treated lungs of C57BL/6 mice were fixed in acetone and examined by immunofluorescence with anti-NG2 antibody (left), FITC-conjugated anti-CD146 antibody (middle), and both images were merged into 1 image (right). CD146 was expressed in NG2-positive pericytes as well as in endothelial cells.

**(C)** Snap-frozen saline-treated lungs of C57BL/6 mice were fixed in acetone and examined by immunofluorescence with anti-α-SMA antibody (left), FITC-conjugated anti-CD146 antibody (middle), and both images were merged into 1 image with a DAPI-stained image (right). CD146 was expressed in α-SMA-positive vascular smooth muscle cells of the pulmonary artery (asterisk) and bronchial smooth muscle cells (arrow).

Figures in (A), (B), and (C) show representative results. Scale bars of (A) and (C) indicate 200 µm. Scale bars of (B) indicate 400 µm.

**Additional file 1: Figure S2**

**CD146 is a lineage-specific cell surface marker of NG2-positive pericytes and vascular smooth muscle cells**

By using flow cytometry, we investigated whether CD146 was expressed on the surface of cells expressing NG2 in lungs of NG2DsRedBAC transgenic mice. Single cells of lungs of C57BL/6 mice were incubated with FITC-conjugated isotype control antibody and PE-conjugated isotype control antibody (left). Single cells of lungs of NG2DsRedBAC transgenic mice were incubated with FITC-conjugated anti-CD146 antibody (right). CD146 was expressed on the surface of most NG2-expressing cells, i.e., pericytes and vascular smooth muscle cells (circle in the right figure). Figures show representative results.

**Additional file 1: Figure S3**

**FACS gating strategy for obtaining single cells of lin^neg^ cells of C57BL/6 lungs**

In the first 3 plots, debris and doublets were excluded by sequential gating on SSC-A vs. FSC-A, followed by FSC-W vs. FSC-H and SSC-W vs. SSC-H. Single cells were plotted for APC-conjugated anti-CD31, CD45, EpCAM, and TER119 antibodies vs. FITC-conjugated anti-CD146 and Lyve-1 antibodies. Lin^neg^ cells were sorted from the APC and FITC double-negative fraction as shown in the rectangle. Figures show representative results.

**Additional file 1: Figure S4**

**CD49e and Sca-1 are not expressed in mesothelial cells**

**(A)** Snap-frozen bleomycin-injured lungs of day 12 were fixed in acetone and examined by DAPI staining (green, left) and immunofluorescence with anti-CD49e antibody (middle), and both images were merged into 1 image (right). Arrows indicate mesothelial cells. Figures show representative results. Scale bars indicate 50 µm.

**(B)** Snap-frozen bleomycin-injured lungs of day 12 were fixed in acetone and examined by DAPI staining (green, left) and immunofluorescence with PE-conjugated anti-Sca-1 antibody (middle), and both images were merged into 1 image (right). Arrows indicate mesothelial cells. Figures show representative results. Scale bars indicate 50 µm.

Immunofluorescence analysis using anti-CD49e antibody and anti-Sca-1 antibody showed that CD49e (A) and Sca-1 (B) were not expressed in mesothelial cells.

**Additional file 1: Figure S5**

**Expression levels of α-SMA, Col1A1, CD49e, lineage-specific cell surface markers in myofibroblasts in IPF lung**

Lung tissues excised from a patient with idiopathic pulmonary fibrosis were fixed in formalin. Immunohistochemical analysis using anti-α-SMA antibody, anti-Col1A1 antibody, anti-CD49e antibody, and antibodies to lineage-specific cell surface markers (CD31, CD45, CD146, EpCAM) was performed. A Fibroblastic focus (dotted line in A) was stained by HE **(A)**, anti-α-SMA antibody **(B)**, anti-Col1A1 antibody **(C)**, anti-CD49e antibody **(D)**, and antibodies to lineage-specific cell surface markers (CD31, CD45, CD146, and EpCAM) **(E)**. A fibrotic scar was stained by HE **(F)**, anti-α-SMA antibody **(G)**, anti-Col1A1 antibody **(H)**, anti-CD49e antibody **(I)**, and antibodies to lineage-specific cell surface markers (CD31, CD45, CD146, and EpCAM) **(J)**. Figures show representative results. Scale bars in figures indicate 200 µm.

**Additional file 1: Figure S6**

**Expression levels of P4ha3 are increased in many types of cells in bleomycin-injured lungs**

To validate the results of qRT-PCR, immunohistochemical analysis using anti-P4ha3 antibody was performed for formalin-fixed saline-treated lungs (left) and bleomycin-injured lungs of day 12 (middle and right). The expression levels of P4ha3 were markedly increased in both fibrotic and non-fibrotic areas of bleomycin-injured lungs. Figures show representative results. Scale bars indicate 200 µm.

**Additional file 1: Figure S1**

**
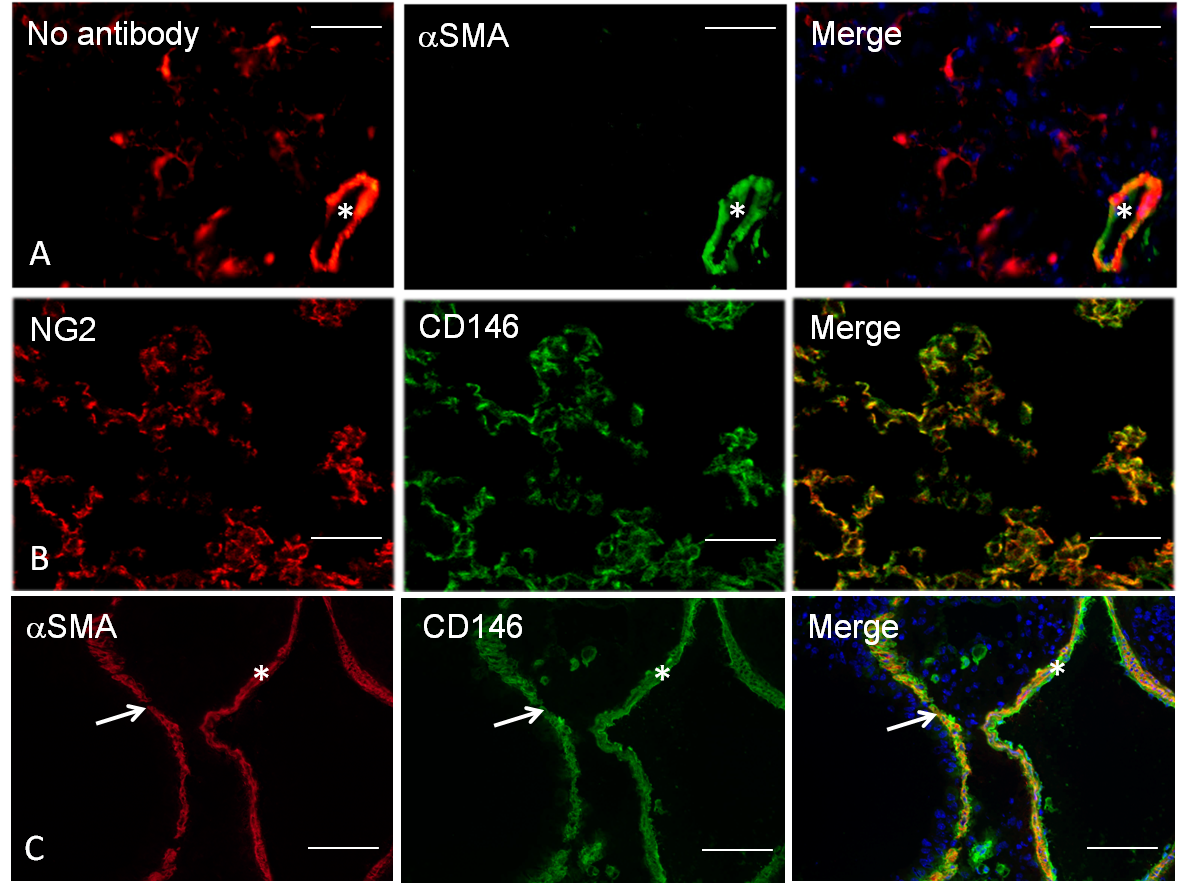
**

**Additional file 1: Figure S2**

**
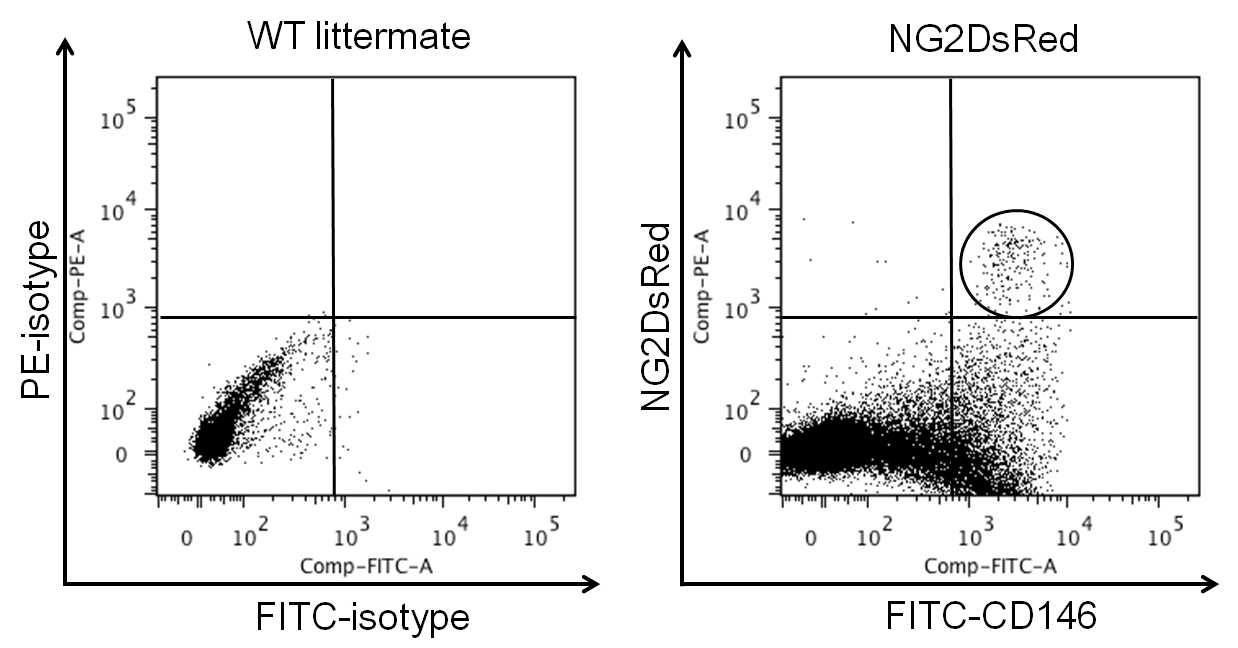
**

**Additional file 1: Figure S3**

**
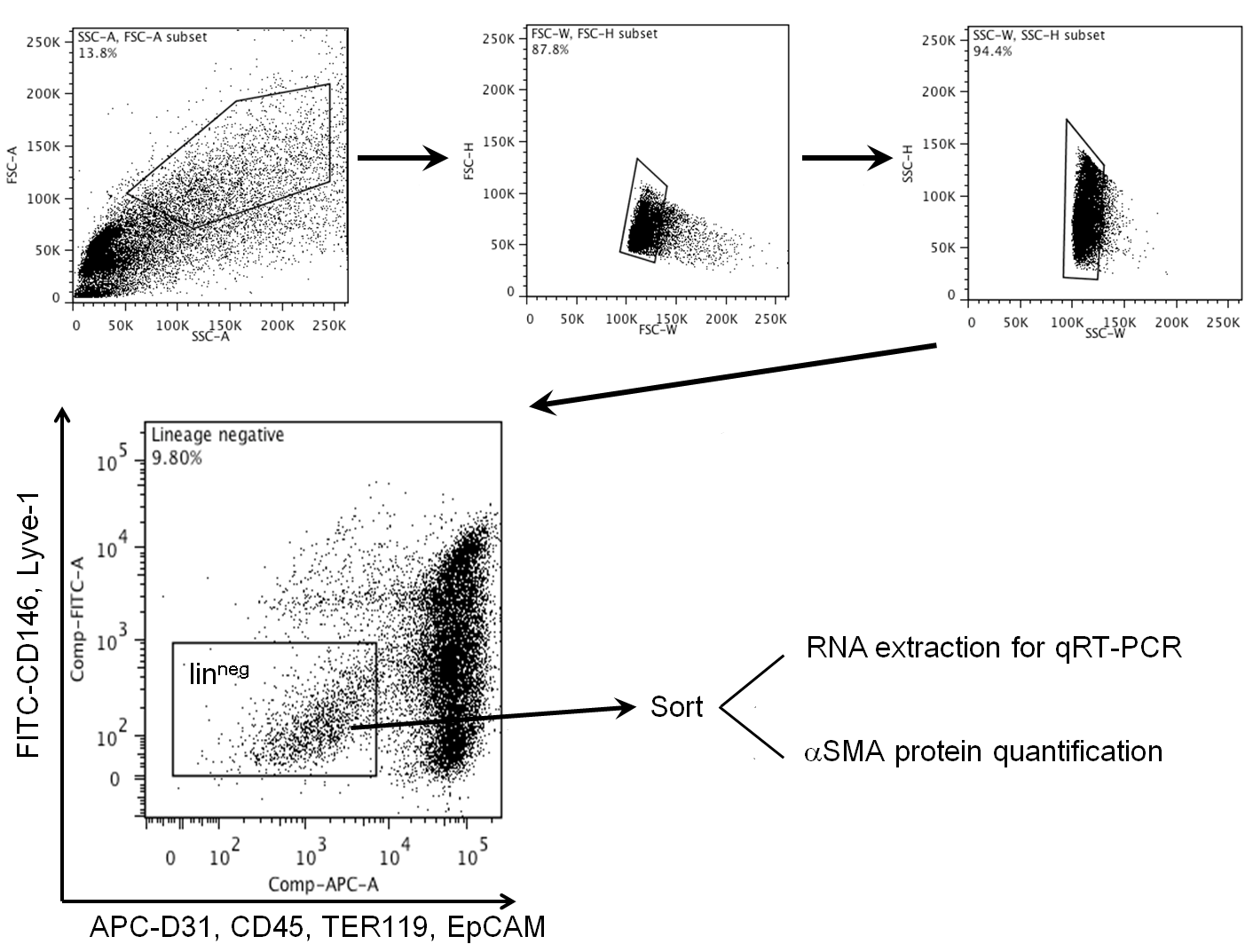
**

**Additional file 1: Figure S4**

**
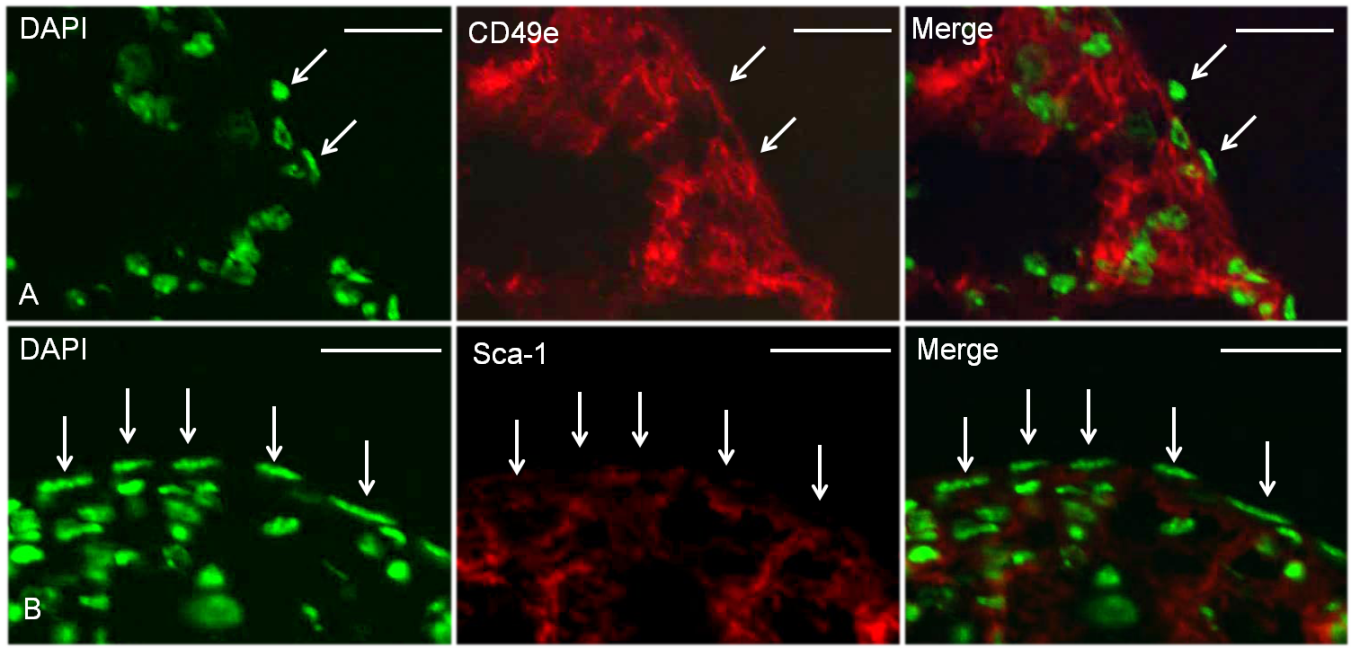
**

**Additional file 1: Figure S5**

**
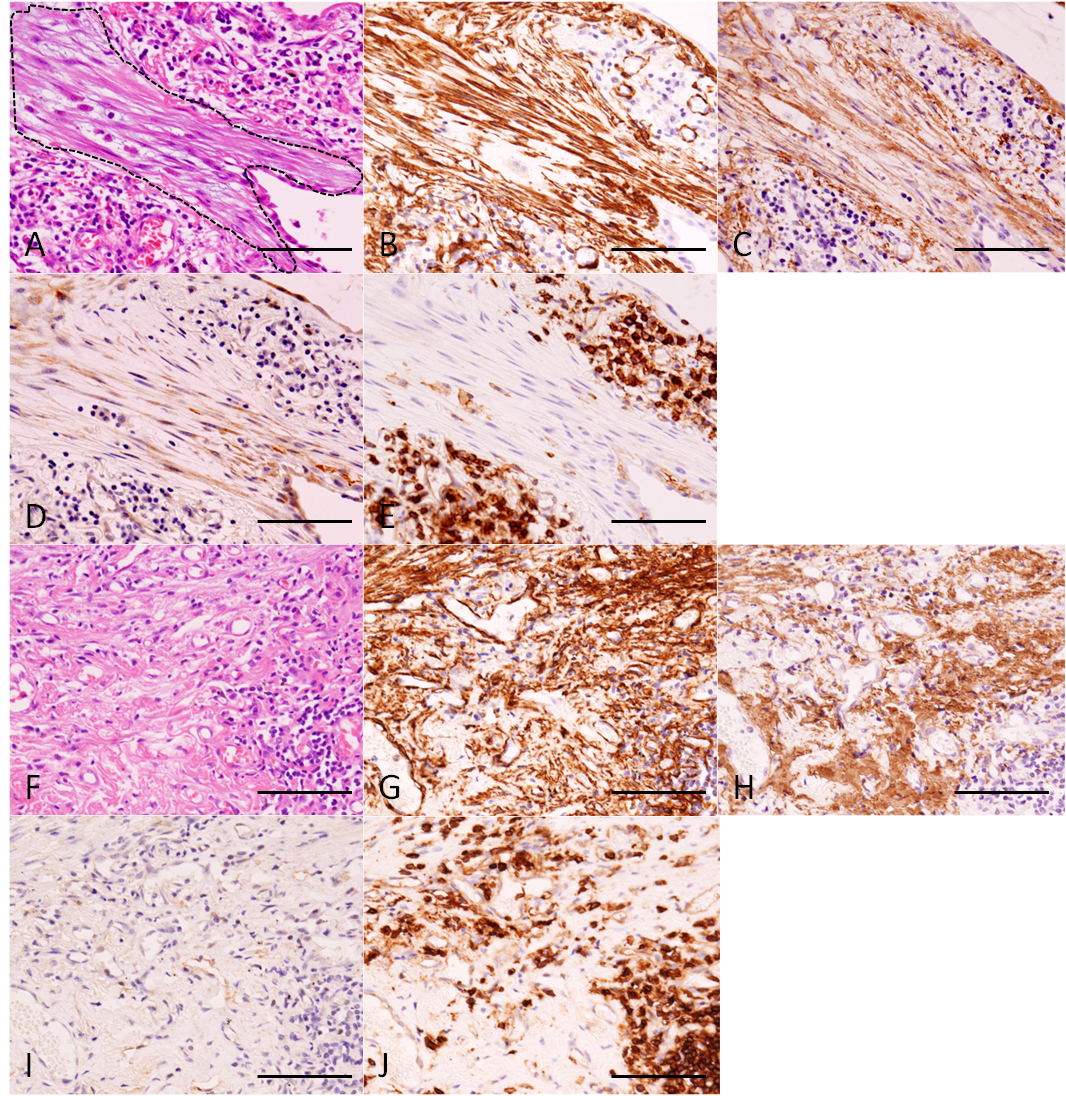
**

**Additional file 1: Figure S6**

**
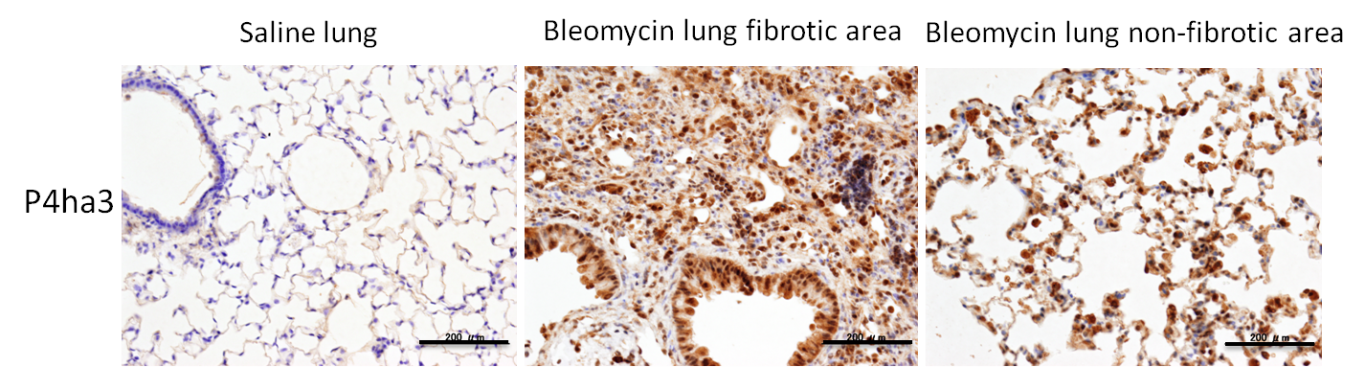
**

**Additional file 1: Table S1**

Information of antibodies used in this study.

| Primary antibody | Fluorescence | Clone | Reactivity | Species | Company | Application | Dilution |
| --- | --- | --- | --- | --- | --- | --- | --- |
| Anti-CD31 (PECAM-1) | APC | 390 | Mouse | Rat monoclonal | eBioscience | FACS | 100 |
| Anti-CD31 (PECAM-1) | FITC | 390 | Mouse | Rat monoclonal | eBioscience | IF | 100 |
| Anti-CD45 | APC | 30-F11 | Mouse | Rat monoclonal | eBioscience | FACS | 100 |
| Anti-CD45 | FITC | 30-F11 | Mouse | Rat monoclonal | eBioscience | IF | 100 |
| Anti-CD49e (Integrinα5) | biotin | 5H10-27 | Mouse | Rat monoclonal | BioLegend | FACS, IF | 150 |
| Anti-CD146 | FITC | ME-9F1 | Mouse | Rat monoclonal | Miltenyi Biotechnology | FACS, IF | 10 |
| Anti-CD326 (EpCAM) | APC | G8.8 | Mouse | Rat monoclonal | BioLegend | FACS | 100 |
| Anti-CD326 (EpCAM) | FITC | G8.8 | Mouse | Rat monoclonal | BioLegend | IF | 100 |
| Anti-Lyve-1 | FITC | ALY7 | Mouse | Rat monoclonal | eBioscience | FACS, IF | 100 |
| Anti-Ly-76 (TER119) | APC | TER119 | Mouse | Rat monoclonal | Biolegend | FACS | 100 |
| Anti-Ly-76 (TER119) | FITC | TER119 | Mouse | Rat monoclonal | Biolegend | IF | 100 |
| Anti-Ly-6A/E (Sca-1) | PE-Cy7 | D7 | Mouse | Rat monoclonal | eBioscience | FACS | 600 |
| Anti-Ly-6A/E (Sca-1) | APC or PE | D7 | Mouse | Rat monoclonal | eBioscience | IF | 200 |
| Anti-αSMA | FITC | 1A4 | Mouse | Mouse IgG_2a_ monoclonal | Abcam | FCM | 50 |
| Anti-αSMA | no | 1A4 | Mouse | Mouse IgG_2a_ monoclonal | Sigma-Aldrich | IF, IHC, ICC | 200 |
| Anti-vimentin | no | EPR3776 | Mouse | Rabbit monoclonal | Epitomics | FCM, ICC | 200 |
| Anti-collagen 1A1 | no | 3G3 | Mouse | Mouse IgG_3_ monoclonal | Sigma-Aldrich | IHC, IF | 100 |
| Anti-NG2 | no |  | Mouse | Rabbit polyclonal | Millipore | IF | 100 |
| Anti-P4ha3 | no |  | Mouse | Rabbit polyclonal | Abcam | IHC | 100 |
| Anti-CD31 (PECAM-1) | no | JC70A | Human | Mouse monoclonal | Dako | IHC | 500 |
| Anti-CD45 | no | M0701 | Human | Mouse monoclonal | Dako | IHC | 500 |
| Anti-CD49e (Integrinα5) | no |  | Human | Rabbit polyclonal | Sigma-Aldrich | IHC | 150 |
| Anti-CD146 | no | NCL-CD146 | Human | Mouse monoclonal | Novocastra | IHC | 50 |
| Anti-EpCAM | no | 1144-1 | Human | Rabbit monoclonal | Epitomics | IHC | 50 |
|  |  |  |  |  |  |  |  |
| Secondary antibody | Fluorescence | Clone | Reactivity | Species | Company | Application | Dilution |
| Anti-mouse IgG_1_ | Alexa fluor 488 |  | Mouse | Goat | Molecular Probes | IF | 200 |
| Anti-mouse IgG_2a_ | Alexa fluor 488 |  | Mouse | Goat | Molecular Probes | IF | 200 |
| Anti-mouse IgG_2a_ | Alexa fluor 546 |  | Mouse | Goat | Molecular Probes | IF | 500 |
| Anti-mouse IgG_3_ | Alexa fluor 594 |  | Mouse | Goat | Molecular Probes | IF | 500 |
| Anti-rabbit IgG | Alexa fluor 488 |  | Rabitt | Goat | Molecular Probes | IF | 200 |
| Anti-rabbit IgG | Alexa fluor 546 |  | Rabitt | Goat | Molecular Probes | IF | 500 |
| Anti-mouse IgG | Peroxidase |  | Mouse | Goat | Nichirei Bioscience | IHC | 1 |
| Anti-rabbit IgG | Peroxidase |  | Rabitt | Goat | Nichirei Bioscience | IHC | 1 |

FACS (fluorescence-activated cell sorting) IF (immunofluorescence)

FCM (flowcytometry) IHC (immunohistochemistry)

ICC (immunocytochemistry)

**Additional file 1: Table S2**

Information of GeneBank accession number, primer sequences, and PCR product size of 114 genes for cell surface markers.

| Suface markers | Definition | GeneBank | 5' primer | 3' primer | Product size |
| --- | --- | --- | --- | --- | --- |
| *CD9* | CD9 antigen. | NM_007657 | ttggtgatattcgccattga | ggttcatccttgctccgtaa | 125 |
| *CD13* | alanyl (membrane) aminopeptidase (Anpep). | NM_008486 | acagttccggaatgcaactc | ggatgtagtccgggttcaga | 122 |
| *CD14* | CD14 antigen. | NM_009841 | tggcccagtcagctaaactc | agggttcctatccagcctgt | 120 |
| *CD16* | Fc fragment of IgG, low affinity IIIa, receptor. | NM_144559 | ggctggctattgcttcagac | tgccgttctgtgaataggtg | 118 |
| *CD18* | integrin beta 2 (Itgb2). | NM_008404 | tctgcagtaatggagcatcg | tcctggatacactcggaagc | 115 |
| *CD21* | complement receptor 2 (Cr2). | NM_007758 | aggatgacagccaatggaac | cagaagccgacactcatcaa | 118 |
| *CD22* | CD22 antigen. | NM_009845 | cgtctgggtcatggaaagat | ccagggtcacactgtctcct | 121 |
| *CD23* | mRNA for Fc E receptor II (FcERII/CD23, pERB452). | X64223 | gcgactagtcagcatccaca | ccgtccgaccatacaaactc | 123 |
| *CD24* | CD24a antigen. | NM_009846 | tggtctcctggctctctctc | cacattggacttgtggttgc | 121 |
| *CD25* | interleukin 2 receptor, alpha chain (Il2ra). | NM_008367 | cccgagagtgagacttcctg | agctggccactgctacctta | 118 |
| *CD26* | dipeptidylpeptidase 4 (Dpp4). | NM_010074 | cactgcagtaccccaagaca | gagccgcactagaggatgag | 117 |
| *CD27* | tumor necrosis factor receptor superfamily, member 7. | NM_001033126 | ttcttatccgcaactgcaca | gtctggtcagtgcagggttt | 120 |
| *CD28* | CD28 antigen. | NM_007642 | tgtcgggaatgggaatttta | ttgacgtgcagattccagag | 117 |
| *CD29* | integrin beta 1 (fibronectin receptor beta) (Itgb1). | NM_010578 | aactgcaccagcccatttag | ccaccttctggagaatccaa | 119 |
| *CD30* | tumor necrosis factor receptor superfamily, member 8. | NM_009401 | catggtggtgctactggttg | gctggaaggtctgcactagg | 113 |
| *CD34* | CD34 antigen. | NM_133654 | cagttggagccctacaggag | attggcctttccctgagtct | 118 |
| *CD36* | CD36 antigen. | NM_007643 | aaaccagtgctctcccttga | tgagaatgcctccaaacaca | 117 |
| *CD38* | CD38 antigen. | NM_007646 | acgccccacttgttaaattg | tccagggtgaacatctttcc | 121 |
| *CD40* | CD40 antigen, transcript variant 1. | NM_011611 | acagggagattcgctgtcac | ggtgcagtgttgtccttcct | 122 |
| *CD43* | sialophorin (Spn), transcript variant 1. | NM_009259 | agtctgcagaggacgacgat | ctccttcgagtccactgtcc | 120 |
| *CD44* | CD44 antigen, transcript variant 1. | NM_009851 | gtgggcagaagaaaaagctg | ttgttcaccaaatgcaccat | 118 |
| *CD45* | protein tyrosine phosphatase, receptor type, C. | NM_011210 | atcatcgccagcatctatcc | ctggacggacacagttagca | 121 |
| *CD47* | CD47 antigen. | NM_010581 | gatttcaggtttggggatca | ccgtcacttcccttcaccta | 119 |
| *CD48* | CD48 antigen. | NM_007649 | aagaaggcaacttggaagca | caaactctcgcagacgttca | 121 |
| *CD49a* | integrin alpha 1 (Itga1). | NM_001033228 | accagtcagcagcttcgttt | gaccacagttccgttccagt | 119 |
| *CD49b* | integrin alpha 2 (Itga2). | NM_008396 | ggttggttcatcgcagaaat | cctggttctggaggttttga | 119 |
| *CD49d* | integrin alpha 4 (Itga4). | NM_010576 | gtactggactggcaccgtct | ccagctccaactgagtagcc | 117 |
| *CD49e* | integrin alpha 5 (fibronectin receptor alpha) (Itga5), | NM_010577 | atatctgccagcgcatctct | aggcattgaggcagaagcta | 118 |
| *CD49f* | integrin alpha 6 (Itga6). | NM_008397 | cgggaacttcctgaaaaaca | ggcacctgatgttcacacac | 115 |
| *CD51* | integrin alpha V (Itgav). | NM_008402 | ggggatgattacgcagatgt | tggaagtctcccactgctct | 122 |
| *CD53* | CD53 antigen. | NM_007651 | ggcaaaatcgtggtttcact | gcctggcttgttttgtcaat | 129 |
| *CD54* | intercellular adhesion molecule (Icam1). | NM_010493 | taagaggactcggtggatgg | gcaggggcaatagagaatga | 112 |
| *CD55* | decay accelerating factor 2 (Daf2). | NM_007827 | cttgggcagacactccaagt | gaccattggccattttcaag | 120 |
| *CD61* | integrin beta 3 (Itgb3). | NM_016780 | catctctggggctgatgact | actgtggtcccaggaatgag | 121 |
| *CD62E* | selectin, endothelial cell (Sele). | NM_011345 | atctggcatctgggataacg | acgcaagttctccagctgtt | 116 |
| *CD62L* | selectin, lymphocyte (Sell). | NM_011346 | ccatgaactgggaaaatgct | aataagggcttttgggcaat | 117 |
| *CD62P* | selectin, platelet (Selp). | NM_011347 | agctgcaatgtttggcttct | cagcaattgggtgcatacag | 120 |
| *CD69* | CD69 antigen. | NM_001033122 | acatctggagagagggcaga | aaggacgtgatgaggaccac | 120 |
| *CD70* | tumor necrosis factor (ligand) superfamily, member 7. | NM_011617 | tctcacagttcctcggaagg | aggccatcttgatggatacg | 120 |
| *CD71* | transferrin receptor (Tfrc). | NM_011638 | tctggaatcccagcagtttc | accatttggttgagctgagg | 119 |
| *CD72* | CD72 antigen. | NM_007654 | cgaagaccaaggagaacctg | ggacagcaggtgtctgatga | 121 |
| *CD73* | 5' nucleotidase, ecto (Nt5e). | NM_011851 | tccaccttccaaagaagtgc | aaccttcaggtagcccaggt | 121 |
| *CD80* | CD80 antigen. | NM_009855 | gatgctcacgtgtcagagga | caacgatgacgacgactgtt | 124 |
| *CD81* | CD81 antigen. | NM_133655 | gtagctctgtggttgcgtca | gctcccacagcaatgagaat | 119 |
| *CD86* | CD86 antigen. | NM_019388 | accgttgtgtgtgttctgga | ggagggccacagtaactgaa | 127 |
| *CD88* | complement component 5a receptor 1 (C5ar1). | NM_007577 | ggtctctccccagcatcata | ctacaccgcctgactcttcc | 116 |
| *CD90* | thymus cell antigen 1, theta (Thy1). | NM_009382 | ctccctccatgcataccact | ctacccaccatacgccctta | 123 |
| *CD93* | CD93 antigen. | NM_010740 | gtggtggccatctcactctt | atagctgtcggctgcattct | 117 |
| *CD94* | killer cell lectin-like receptor, subfamily D, member. | NM_010654 | tcggtggagactgatgtctg | agtggtggttggagaaggtg | 127 |
| *CD95* | Fas (TNF receptor superfamily member) (Fas). | NM_007987 | atgcacactctgcgatgaag | cagtgttcacagccaggaga | 120 |
| *CD98* | solute carrier family 3. | NM_008577 | acctggtggtgctcaacttc | gggcactgtcggtactaagc | 120 |
| *CD100* | sema domain, immunoglobulin domain (Ig), transmembrane. | NM_013660 | tggagcagtctcacaccaag | ctgggagattcaaggagctg | 120 |
| *CD102* | intercellular adhesion molecule 2 (Icam2). | NM_010494 | gtggcagagagaccctgaag | tggcaggaaaagttgagacc | 118 |
| *CD103* | integrin, alpha E, epithelial-associated (Itgae). | NM_008399 | ccctggaccactacaaggaa | cagttcctgctgggagatgt | 122 |

| Suface markers | Definition | GeneBank | 5' primer | 3' primer | Product size |
| --- | --- | --- | --- | --- | --- |
| *CD104* | integrin beta 4 (Itgb4), transcript variant 1. | NM_001005608 | ggagactgggtcctttcaca | cccccttggtcttctttagc | 120 |
| *CD105* | endoglin (Eng). | NM_007932 | cttccaaggacagccaagag | gggtcatccagtgctgctat | 120 |
| *CD106* | vascular cell adhesion molecule 1 (Vcam1). | NM_011693 | cccaaacagaggcagagtgt | tgagcaggtcaggttcacag | 120 |
| *CD107a* | lysosomal membrane glycoprotein 1 (Lamp1). | NM_010684 | cccacaaaccccactgtatc | ggtcaccgtcttgttgtcct | 120 |
| *CD107b* | lysosomal membrane glycoprotein 2 (Lamp2), transcript. | NM_001017959 | aggctgaacaacagccaaat | aagctgagccattagccaaa | 115 |
| *CD115* | colony stimulating factor 1 receptor (Csf1r). | NM_001037859 | atgacacccaccctgaagtc | cacactgttgtgggttttgc | 122 |
| *CD117* | kit oncogene (Kit). | NM_021099 | tcaaaggaaatgcacgactg | aggagaagagctcccagagg | 120 |
| *CD119* | interferon gamma receptor 1 (Ifngr1). | NM_010511 | ttcaccctgaagtcgttgtg | gatctccccactccggttat | 116 |
| *CD120a* | tumor necrosis factor receptor superfamily, member 1a. | NM_011609 | cagtctgcagggagtgtgaa | tcagcttggcaaggagagat | 124 |
| *CD121a* | interleukin 1 receptor, type I (Il1r1). | NM_008362 | ggttgattgcagaaccacct | ccttgtcaggtggcagaaat | 120 |
| *CD121b* | interleukin 1 receptor, type II (Il1r2). | NM_010555 | gaggggctacaccaccagta | ggattcgaggcaacacattt | 119 |
| *CD122* | interleukin 2 receptor, beta chain (Il2rb). | NM_008368 | gaagtgctcgacggagattc | gaagtagccctggttggtga | 120 |
| *CD123* | interleukin 3 receptor, alpha chain (Il3ra). | NM_008369 | tgacctcgacactgtccttg | gtccacagtgttgtccatgc | 121 |
| *CD124* | interleukin 4 receptor, alpha (Il4ra). | NM_001008700 | gtcacagagcagccttcaca | aaaactccggtaggcaggat | 120 |
| *CD126* | interleukin 6 receptor, alpha (Il6ra). | NM_010559 | cctgccaaccttgtggtatc | tcggtatcgaagctggaact | 120 |
| *CD127* | interleukin 7 receptor (Il7r). | NM_008372 | tttctgcccaatgatcttcc | actgtttctggtgggctgac | 116 |
| *CD133* | prominin 1 (Prom1). | NM_008935 | tcaaagggacccagaaactg | gccttgttcttggtgttggt | 118 |
| *CD134* | tumor necrosis factor receptor superfamily, member 4. | NM_011659 | cttttctccaggcaacaacc | aggaggcttctgtcctcaca | 120 |
| *CD135* | FMS-like tyrosine kinase 3 (Flt3). | NM_010229 | gaaaatgatgacgcccagtt | gtagcgggtagccatcagag | 118 |
| *CD137* | tumor necrosis factor receptor superfamily, member 9. | NM_011612 | gagctaacgaagcagggttg | tcccggtcttaagcacagac | 124 |
| *CD140a* | platelet derived growth factor receptor, alpha. | NM_011058 | agttcctgcatccattttgg | tgcttgcagatcatccagtc | 120 |
| *CD140b* | platelet derived growth factor receptor, beta. | NM_008809 | tgtgatcgagaatggctacg | ggacagaaggcatcggataa | 119 |
| *CD147* | basigin (Bsg). | NM_009768 | cctgcatcttccttcctgag | gaccagtttcgcaagctctc | 119 |
| *CD150* | signaling lymphocytic activation molecule family. | NM_013730 | tcgagtccatggatgcaata | ggctggcagtgatttgattt | 116 |
| *CD152* | cytotoxic T-lymphocyte-associated protein 4 (Ctla4). | NM_009843 | tggactccggaggtacaaag | ctgaaggttgggtcacctgt | 122 |
| *CD153* | tumor necrosis factor (ligand) superfamily, member 8. | NM_009403 | ggcctacctccaagtgtcaa | tacaagccagggaattggac | 120 |
| *CD154* | CD40 ligand (Cd40lg). | NM_011616 | gacctgccctgtgttgaact | ttttgcctgccctgtaattc | 119 |
| *CD157* | bone marrow stromal cell antigen 1 (Bst1). | NM_009763 | accccattcctagggacaag | aatcgccaactttgccatac | 123 |
| *CD162* | selectin, platelet (p-selectin) ligand (Selpl). | NM_009151 | aactactcccccacggagat | ctgggctctgtcttcaggtc | 119 |
| *CD172a* | signal-regulatory protein alpha (Sirpa). | NM_007547 | ggggtcaacatcttccacac | tctttgggcagattcaggtc | 120 |
| *CD178* | Fas ligand (TNF superfamily, member 6) (Fasl). | NM_010177 | catcacaaccactcccactg | tcctaatcccattccaacca | 122 |
| *CD180* | CD180 antigen. | NM_008533 | ttcccctcccttactcacct | caatgtcatcatggctgagg | 118 |
| *CD184* | chemokine (C-X-C motif) receptor 4 (Cxcr4). | NM_009911 | gaaactgctggctgaaaagg | ctgtcatccccctgactgat | 120 |
| *CD193* | chemokine (C-C motif) receptor 3 (Ccr3). | NM_009914 | cttcggctctttttccacag | gatttcttgctccccagttg | 120 |
| *CD195* | chemokine (C-C motif) receptor 5 (Ccr5). | NM_009917 | tgatcgtgcaagctcagtct | ccattcctactcccaagctg | 120 |
| *CD197* | chemokine (C-C motif) receptor 7 (Ccr7). | NM_007719 | aacgggctggtgatactgac | taggcccagaagggaagaat | 122 |
| *CD200* | CD200 antigen. | NM_010818 | agctgggactctggaactca | aaggcaagctgttcctgaga | 119 |
| *CD202b* | endothelial-specific receptor tyrosine kinase (Tek). | NM_013690 | tgtgtctgatgccgaaacat | acctccagtggatcttggtg | 120 |
| *CD206* | ATPas, class II, type 9B (Atp9b). | NM_015805 | ttgccaaaagctcacacttg | tgcacagtcatgcttccttc | 119 |
| *CD207* | CD 207 antigen. | NM_144943 | ggaattccacactggattgg | ttgttgggttcacctggaat | 122 |
| *CD210* | interleukin 10 receptor, alpha (Il10ra). | NM_008348 | cattccagggctaccagaaa | ctgcaggtgtaccccaagtt | 122 |
| *CD212* | interleukin 12 receptor, beta 1 (Il12rb1). | NM_008353 | aacaggacggtatccctgtg | ggtcgtcttggtccagttgt | 119 |
| *CD223* | lymphocyte-activation gene 3 (Lag3). | NM_008479 | tttcctgttactgccccaag | ctccagacccagaaccttga | 118 |
| *CD244* | CD244 natural killer cell receptor 2B4 (Cd244). | NM_018729 | agcagcctgcacacatacac | ccaaagggcagaaatctgaa | 119 |
| *CD252* | tumor necrosis factor (ligand) superfamily, member 4. | NM_009452 | cgatggtcgaaggattgtct | caatcagctccccatcattt | 125 |
| *CD253* | TNF-related apoptosis inducing ligand TRAIL. | U37522 | ggacctcagcttcagtcagc | ctgcttcatctcgttggtga | 121 |
| *CD254* | tumor necrosis factor (ligand) superfamily, member 11. | NM_011613 | gactccatgaaaacgcaggt | tgtgttgcagttccttctgc | 120 |
| *CD262* | KILLER/DR5 TRAIL death-inducing receptor. | AF176833 | tgactacaccagccattcca | ttgcatcgacacaccgtatt | 120 |
| *CD265* | tumor necrosis factor receptor superfamily, member 11a. | NM_009399 | aagacggtgctggagtctgt | agaggtctccttgcgtctca | 114 |
| *CD266* | tumor necrosis factor receptor superfamily, member 12a. | NM_013749 | ccgagccagactctttcaac | ccagagatagggcaattgga | 113 |
| *CD273* | programmed cell death 1 ligand 2 (Pdcd1lg2). | NM_021396 | agtaccgttgcctggtcatc | ctcccctgtacctggaacct | 119 |
| *CD274* | CD274 antigen. | NM_021893 | gcttttgaagggaaatgctg | ttgactttcagcgtgattcg | 120 |
| *CD275* | icos ligand (Icosl). | NM_015790 | gagttcacatgccgggtatt | tcagaggtgctgatgacagg | 116 |
| *CD276* | CD276 antigen. | NM_133983 | ggggctctctgtctgtcttg | tctccatctccatcctggtc | 120 |
| *CD278* | inducible T-cell co-stimulator (Icos). | NM_017480 | tgacccacctccttttcaag | taccacaacgaaagctgcac | 121 |
| *CD279* | programmed cell death 1 (Pdcd1). | NM_008798 | gccctagtgggtatccctgt | gctcctccttcagagtgtcg | 118 |
| *CD282* | toll-like receptor 2 (Tlr2). | NM_011905 | gagtctgctgtgcccttctc | gctttcttgggcttcctctt | 124 |
| *CD283* | toll-like receptor 3 (Tlr3). | NM_126166 | tcggattcttggtttcaagg | tcttccattggggagaaatg | 120 |
| *CD284* | toll-like receptor 4 (Tlr4). | NM_021297 | tcaagaccaagcctttcagg | tcaaccgatggacgtgtaaa | 122 |
| *Sca-1* | lymphocyte antigen 6 complex, locus A (Ly6a), | NM_010738 | tcagtcctcctgcagacctt | actcccaccttggagcttct | 136 |

**Additional file 1: Table S3**

Information of GeneBank accession number, primer sequences, and PCR product size of genes for collagen, collagen synthesis enzymes, and chemokines.

| Gene | Definition | GeneBank | 5' primer | 3' primer | Product size |
| --- | --- | --- | --- | --- | --- |
| *Col1a1* | collagen, type I, alpha 1 | NM_007742 | gagcggagagtactggatcg | gttcgggctgatgtaccagt | 142 |
| *Col1a2* | collagen, type I, alpha 2 | NM_007743 | gtcctagtcgatggctgctc | caatgtccagaggtgcaatg | 115 |
| *Col3a1* | collagen, type III, alpha 1 | NM_009930 | accaaaaggtgatgctggac | gacctcgtgctccagttagc | 110 |
| *P4ha1* | proline 4-hydroxylase, alpha 1 | NM_011030 | tagcaaaaccaaggctgagg | ggtcttcgtagccagacagc | 111 |
| *P4ha2* | proline 4-hydroxylase, alpha 2, transcript variant 1 | NM_001142916 | tcttccccactgatgaggac | agttcccctctggaaatcgt | 100 |
| *P4ha3* | proline 4-hydroxylase, alpha 3 | NM_177161 | cagaaaatccgagagcttgc | tttagccaggcacttttgct | 107 |
| *P4hb* | prolyl 4-hydroxylase, beta | NM_011032 | gttttgccaccgcttcttag | cgccccaaccagtactttta | 103 |
| *Plod1* | procollagen lysine, 2-oxoglutarate 5-dioxygenase 1 | NM_011122 | aatatcagcctggaccatcg | taggccagatttcgtgctct | 110 |
| *Plod2* | procollagen lysine, 2-oxoglutarate 5-dioxygenase 2 | NM_001142916 | ggattttcagggaggtggat | gtccttcgtgcaaatgtgtg | 113 |
| *Plod3* | procollagen lysine, 2-oxoglutarate 5-dioxygenase 3 | NM_011962 | gagcagcctactcccttcct | ggtacacctcgctgttgtga | 103 |
| *Hsp47 (Serpinh1)* | serine (or cysteine) peptidase inhibitor, clade H, member 1 | NM_009825 | caactgcgaacactccaaga | ctccacatccttggtgacct | 118 |
| *Procollagen 1 N proteinase (Adamts2)* | a disintegrin-like and metallopeptidase (reprolysin type) with thrombospondin type 1 motif, 2 (Adamts2) | NM_175643 | cccgatatcctcaaacgaga | gatgtgggttgtcacactgg | 115 |
| *Lox* | lysyl oxidase | NM_010728 | cagaggagagtggctgaagg | ccaggactcaatccctgtgt | 116 |
| *Loxl1* | lysyl oxidase-like 1 | NM_010729 | ctatgcctgcacctctcaca | gtagttcccaggctgcacat | 112 |
| *Loxl2* | lysyl oxidase-like 2 | NM_033325 | aagaattcccacgtggtctg | cttccagtaacgcagcttcc | 108 |
| *Ccl2* | chemokine (C-C motif) ligand 2 | NM_011333 | agcaccagccaactctcact | cgttaactgcatctggctga | 136 |
| *Ccl5* | chemokine (C-C motif) ligand 5 | NM_013653 | atatggctcggacaccactc | tccttcgagtgacaaacacg | 131 |
| *Ccl7* | chemokine (C-C motif) ligand 7 | NM_013654 | aagtgggtcgaggaggctat | agaaagaacagcggtgagga | 127 |
| *Ccl11* | chemokine (C-C motif) ligand 11 | NM_011330 | cacggtcacttccttcacct | gctttcagggtgcatctgtt | 147 |
| *Cxcl1* | chemokine (C-X-C motif) ligand 1 | NM_008176 | cttgaaggtgttgccctcag | tggggacaccttttagcatc | 140 |
| *Cxcl2* | chemokine (C-X-C motif) ligand 2 | NM_009140 | agtgaactgcgctgtcaatg | gcccttgagagtggctatga | 126 |
| *Cxcl5* | chemokine (C-X-C motif) ligand 5 | NM_009141 | gccctacggtggaagtcata | gtgcattccgcttagctttc | 132 |
| *Cxcl10* | chemokine (C-X-C motif) ligand 10 | NM_021274 | aagtgctgccgtcattttct | cctatggccctcattctcac | 129 |
| *Cxcl12* | chemokine (C-X-C motif) ligand 12, transcript variant 1 | NM_021704 | agagccaacgtcaagcatct | taatttcgggtcaatgcaca | 108 |
| *Cxcl14* | chemokine (C-X-C motif) ligand 14 | NM_019568 | tatcgtcaccaccaagagca | cttctcgttccaggcattgt | 112 |
| *Cx3cl1* | chemokine (C-X3-C motif) ligand 1 | NM_009142 | ggctaagcctcagagcattg | ctgtagtggagggggactca | 134 |
| *Actb* | actin, beta | NM_007393 | tacagcttcaccaccacagc | tctccagggaggaagaggat | 121 |
| *18s rRNA* | 18S ribosomal RNA | NR_003278 | aaacggctaccacatccaag | cctccaatggatcctcgtta | 155 |
| *Acta2* | actin, alpha 2, smooth muscle, aorta (alpha smooth muscle actin) | NM_007392 | tgtgctggactctggagatg | gaaggaatagccacgctcag | 148 |

**Additional file 1: Table S4**

Raw data of qRT-PCR of genes for collagen and collagen synthesis enzymes of fibroblasts of saline-treated lungs (S Fibro), fibroblasts of bleomycin-injured lungs (B Fibro), myofibroblasts (Myo), and cultured myofibroblast-like cells (TGF+) at three independent experiments.

|  |  |  |  |  | Relative expression to average value of Δct of N fibro | | |  |  | Log_2_ transform of relative expression to average value of Δct of N fibro | | |
| --- | --- | --- | --- | --- | --- | --- | --- | --- | --- | --- | --- | --- |
| Sample Name | Gene | Δct experiment1 | Δct  experiment2 | Δct experiment3 | Δct experiment1 | Δct  experiment2 | Δct experiment3 | **Average of** Δ**ct** | s.d. of Δct | Δct experiment1 | Δct experiment2 | Δct experiment3 |
| S Fibro | *Col1a1* | 3.77 | 2.97 | 2.89 | 0.70 | 1.12 | 1.18 | **1** | 0.26 | -0.51 | 0.17 | 0.24 |
| B Fibro | *Col1a1* | 1.14 | 1.51 | 1.43 | 3.29 | 2.65 | 2.78 | **2.91** | 0.34 | 1.72 | 1.41 | 1.48 |
| Myo | *Col1a1* | 0.77 | 0.66 | 1.25 | 4.09 | 4.37 | 3.09 | **3.85** | 0.67 | 2.03 | 2.13 | 1.63 |
| TGF+ | *Col1a1* | 2.43 | 3.02 | 3.02 | 1.55 | 1.09 | 1.09 | **1.24** | 0.26 | 0.63 | 0.13 | 0.12 |
| S Fibro | *Col1a2* | 5.06 | 4.25 | 4.50 | 0.75 | 1.21 | 1.04 | **1** | 0.23 | -0.42 | 0.27 | 0.06 |
| B Fibro | *Col1a2* | 3.12 | 3.49 | 3.54 | 2.34 | 1.89 | 1.84 | **2.02** | 0.28 | 1.23 | 0.92 | 0.88 |
| Myo | *Col1a2* | 3.03 | 2.88 | 3.70 | 2.48 | 2.71 | 1.67 | **2.29** | 0.54 | 1.31 | 1.44 | 0.74 |
| TGF+ | *Col1a2* | 5.23 | 5.93 | 6.04 | 0.68 | 0.45 | 0.42 | **0.52** | 0.14 | -0.56 | -1.16 | -1.25 |
| S Fibro | *Col3a1* | 5.71 | 4.68 | 4.31 | 0.59 | 1.07 | 1.34 | **1** | 0.38 | -0.77 | 0.10 | 0.42 |
| B Fibro | *Col3a1* | 2.92 | 3.27 | 2.86 | 3.02 | 2.46 | 3.14 | **2.87** | 0.36 | 1.59 | 1.30 | 1.65 |
| Myo | *Col3a1* | 4.01 | 4.01 | 3.98 | 1.59 | 1.59 | 1.62 | **1.60** | 0.02 | 0.67 | 0.67 | 0.70 |
| TGF+ | *Col3a1* | 8.30 | 8.75 | 9.00 | 0.13 | 0.10 | 0.09 | **0.10** | 0.02 | -2.96 | -3.35 | -3.56 |
| S Fibro | *P4ha1* | 11.05 | 10.57 | 10.95 | 0.89 | 1.17 | 0.94 | **1** | 0.15 | -0.17 | 0.23 | -0.09 |
| B Fibro | *P4ha1* | 10.11 | 10.96 | 11.20 | 1.54 | 0.93 | 0.81 | **1.10** | 0.39 | 0.62 | -0.10 | -0.30 |
| Myo | *P4ha1* | 9.93 | 9.99 | 10.72 | 1.72 | 1.66 | 1.08 | **1.48** | 0.35 | 0.78 | 0.73 | 0.11 |
| TGF+ | *P4ha1* | 9.42 | 9.75 | 8.99 | 2.31 | 1.91 | 2.98 | **2.40** | 0.54 | 1.21 | 0.93 | 1.57 |
| S Fibro | *P4ha2* | 11.19 | 9.77 | 9.75 | 0.53 | 1.23 | 1.24 | **1** | 0.41 | -0.91 | 0.30 | 0.31 |
| B Fibro | *P4ha2* | 10.16 | 10.55 | 10.03 | 0.98 | 0.78 | 1.06 | **0.94** | 0.14 | -0.03 | -0.37 | 0.08 |
| Myo | *P4ha2* | 8.80 | 7.94 | 8.93 | 2.17 | 3.60 | 2.01 | **2.59** | 0.88 | 1.12 | 1.85 | 1.01 |
| TGF+ | *P4ha2* | 9.37 | 9.01 | 9.05 | 1.55 | 1.92 | 1.87 | **1.78** | 0.20 | 0.64 | 0.94 | 0.91 |
| S Fibro | *P4ha3* | 14.76 | 13.50 | 14.01 | 0.64 | 1.36 | 1.00 | **1** | 0.36 | -0.64 | 0.44 | 0.001 |
| B Fibro | *P4ha3* | 9.72 | 10.04 | 9.94 | 12.48 | 10.36 | 10.99 | **11.28** | 1.09 | 3.64 | 3.37 | 3.46 |
| Myo | *P4ha3* | 8.72 | 9.01 | 9.00 | 22.47 | 18.93 | 19.03 | **20.15** | 2.02 | 4.49 | 4.24 | 4.25 |
| TGF+ | *P4ha3* | 11.43 | 11.73 | 11.03 | 4.56 | 3.83 | 5.79 | **4.73** | 0.99 | 2.19 | 1.94 | 2.53 |
| S Fibro | *P4hb* | 8.74 | 8.25 | 8.31 | 0.83 | 1.11 | 1.07 | **1** | 0.15 | -0.27 | 0.15 | 0.09 |
| B Fibro | *P4hb* | 7.05 | 7.50 | 7.45 | 2.23 | 1.72 | 1.76 | **1.90** | 0.29 | 1.16 | 0.78 | 0.82 |
| Myo | *P4hb* | 6.69 | 6.77 | 7.17 | 2.75 | 2.64 | 2.09 | **2.49** | 0.36 | 1.46 | 1.40 | 1.06 |
| TGF+ | *P4hb* | 7.42 | 7.72 | 7.01 | 1.80 | 1.51 | 2.29 | **1.86** | 0.39 | 0.85 | 0.59 | 1.19 |
| S Fibro | *Plod1* | 10.78 | 10.14 | 9.72 | 0.69 | 1.01 | 1.29 | **1** | 0.30 | -0.53 | 0.02 | 0.37 |
| B Fibro | *Plod1* | 8.48 | 9.11 | 8.50 | 2.67 | 1.85 | 2.65 | **2.39** | 0.47 | 1.42 | 0.88 | 1.41 |
| Myo | *Plod1* | 8.61 | 9.01 | 8.76 | 2.48 | 1.96 | 2.27 | **2.24** | 0.26 | 1.31 | 0.97 | 1.18 |
| TGF+ | *Plod1* | 10.10 | 9.99 | 9.81 | 1.03 | 1.10 | 1.22 | **1.12** | 0.10 | 0.05 | 0.14 | 0.29 |
| S Fibro | *Plod2* | 11.73 | 11.16 | 11.49 | 0.85 | 1.18 | 0.97 | **1** | 0.17 | -0.24 | 0.24 | -0.04 |
| B Fibro | *Plod2* | 9.72 | 10.26 | 10.30 | 2.75 | 2.01 | 1.96 | **2.24** | 0.44 | 1.46 | 1.01 | 0.97 |
| Myo | *Plod2* | 9.42 | 9.74 | 9.83 | 3.29 | 2.73 | 2.59 | **2.87** | 0.37 | 1.72 | 1.45 | 1.37 |
| TGF+ | *Plod2* | 9.41 | 9.70 | 9.00 | 3.31 | 2.78 | 4.22 | **3.44** | 0.73 | 1.73 | 1.48 | 2.08 |
| S Fibro | *Plod3* | 11.74 | 10.59 | 10.83 | 0.64 | 1.26 | 1.10 | **1** | 0.32 | -0.64 | 0.34 | 0.13 |
| B Fibro | *Plod3* | 10.89 | 10.49 | 10.56 | 1.06 | 1.34 | 1.29 | **1.23** | 0.15 | 0.08 | 0.42 | 0.36 |
| Myo | *Plod3* | 10.03 | 9.54 | 9.90 | 1.75 | 2.34 | 1.89 | **1.99** | 0.31 | 0.81 | 1.23 | 0.92 |
| TGF+ | *Plod3* | 10.10 | 10.50 | 10.95 | 1.69 | 1.33 | 1.02 | **1.35** | 0.33 | 0.75 | 0.41 | 0.03 |
| S Fibro | *Hsp47* | 6.70 | 6.46 | 5.94 | 0.81 | 0.93 | 1.26 | **1** | 0.24 | -0.31 | -0.11 | 0.34 |
| B Fibro | *Hsp47* | 6.14 | 6.49 | 6.41 | 1.12 | 0.91 | 0.96 | **1.00** | 0.11 | 0.17 | -0.13 | -0.06 |
| Myo | *Hsp47* | 5.35 | 5.45 | 5.74 | 1.78 | 1.68 | 1.42 | **1.63** | 0.19 | 0.83 | 0.75 | 0.50 |
| TGF+ | *Hsp47* | 5.81 | 6.01 | 5.57 | 1.36 | 1.21 | 1.56 | **1.38** | 0.18 | 0.44 | 0.28 | 0.65 |
| S Fibro | *Adamts2* | 9.50 | 9.19 | 8.53 | 0.76 | 0.91 | 1.34 | **1** | 0.30 | -0.41 | -0.14 | 0.42 |
| B Fibro | *Adamts2* | 8.14 | 8.45 | 7.93 | 1.68 | 1.40 | 1.90 | **1.66** | 0.25 | 0.75 | 0.49 | 0.93 |
| Myo | *Adamts2* | 9.51 | 9.87 | 9.54 | 0.75 | 0.61 | 0.74 | **0.70** | 0.08 | -0.42 | -0.72 | -0.44 |
| TGF+ | *Adamts2* | 12.44 | 13.02 | 12.91 | 0.13 | 0.10 | 0.10 | **0.11** | 0.02 | -2.90 | -3.39 | -3.30 |
| S Fibro | *Lox* | 9.96 | 9.35 | 9.51 | 0.80 | 1.15 | 1.05 | **1** | 0.18 | -0.32 | 0.20 | 0.07 |
| B Fibro | *Lox* | 6.51 | 6.84 | 6.40 | 6.11 | 5.01 | 6.49 | **5.87** | 0.77 | 2.61 | 2.33 | 2.70 |
| Myo | *Lox* | 6.00 | 5.98 | 6.17 | 8.24 | 8.32 | 7.44 | **8.00** | 0.48 | 3.04 | 3.06 | 2.90 |
| TGF+ | *Lox* | 6.41 | 6.55 | 6.52 | 6.45 | 5.94 | 6.07 | **6.15** | 0.26 | 2.69 | 2.57 | 2.60 |
| S Fibro | *Loxl1* | 8.97 | 8.79 | 9.42 | 1.04 | 1.16 | 0.80 | **1** | 0.18 | 0.06 | 0.21 | -0.32 |
| B Fibro | *Loxl1* | 7.54 | 8.50 | 9.04 | 2.42 | 1.37 | 1.00 | **1.60** | 0.74 | 1.28 | 0.45 | 0.001 |
| Myo | *Loxl1* | 9.03 | 8.55 | 9.73 | 1.01 | 1.34 | 0.67 | **1.00** | 0.34 | 0.01 | 0.42 | -0.59 |
| TGF+ | *Loxl1* | 9.42 | 10.13 | 10.82 | 0.80 | 0.53 | 0.35 | **0.56** | 0.23 | -0.32 | -0.93 | -1.51 |
| S Fibro | *Loxl2* | 10.22 | 9.84 | 9.00 | 0.70 | 0.87 | 1.43 | **1** | 0.38 | -0.52 | -0.20 | 0.51 |
| B Fibro | *Loxl2* | 7.82 | 8.51 | 7.68 | 2.87 | 1.91 | 3.11 | **2.63** | 0.64 | 1.52 | 0.93 | 1.64 |
| Myo | *Loxl2* | 7.03 | 7.00 | 6.54 | 4.55 | 4.63 | 6.07 | **5.08** | 0.85 | 2.19 | 2.21 | 2.60 |
| TGF+ | *Loxl2* | 7.34 | 7.87 | 8.02 | 3.81 | 2.77 | 2.54 | **3.04** | 0.67 | 1.93 | 1.47 | 1.35 |

**Additional file 1: Table S5**

Raw data of qRT-PCR of chemokine genes of fibroblasts of saline-treated lungs (S Fibro), fibroblasts of bleomycin-injured lungs (B Fibro), myofibroblasts (Myo), cultured myofibroblast-like cells (TGF+), unfractionated cells of saline-treated lungs (S unfra), and unfractionated cells of bleomycin-injured lungs (B unfra) at three independent experiments.

|  |  |  |  |  | Relative expression to average value of Δct of S fibro | | |  |  | Log_2_ transform of relative expression to average value of Δct of S fibro | | |
| --- | --- | --- | --- | --- | --- | --- | --- | --- | --- | --- | --- | --- |
| Sample Name | Gene | Δct experiment1 | Δct experiment2 | Δct experiment3 | Δct experiment1 | Δct experiment2 | Δct experiment3 | **Average of** Δ**ct** | s.d. of Δct | Δct experiment1 | Δct experiment2 | Δct experiment3 |
| S Fibro | *Ccl2* | 11.74 | 10.69 | 11.32 | 0.73 | 1.34 | 0.93 | **1** | 0.32 | -0.46 | 0.43 | -0.11 |
| B Fibro | *Ccl2* | 9.87 | 9.66 | 10.03 | 2.18 | 2.46 | 1.98 | **2.21** | 0.24 | 1.12 | 1.30 | 0.99 |
| Myo | *Ccl2* | 9.97 | 10.00 | 11.74 | 2.05 | 2.02 | 0.73 | **1.60** | 0.76 | 1.04 | 1.02 | -0.46 |
| TGF+ | *Ccl2* | 16.07 | 16.72 | 16.80 | 0.06 | 0.04 | 0.04 | **0.04** | 0.01 | -4.13 | -4.68 | -4.75 |
| S unfra | *Ccl2* | 10.04 | 9.98 | 10.59 | 1.97 | 2.05 | 1.43 | **1.81** | 0.34 | 0.98 | 1.03 | 0.51 |
| B unfra | *Ccl2* | 10.84 | 10.79 | 10.96 | 1.23 | 1.27 | 1.15 | **1.22** | 0.06 | 0.30 | 0.35 | 0.20 |
| S Fibro | *Ccl5* | 13.47 | 12.95 | 15.88 | 1.15 | 1.57 | 0.28 | **1** | 0.66 | 0.21 | 0.65 | -1.83 |
| B Fibro | *Ccl5* | 13.54 | 14.49 | 13.04 | 1.11 | 0.64 | 1.49 | **1.08** | 0.43 | 0.15 | -0.65 | 0.58 |
| Myo | *Ccl5* | 15.00 | 15.81 | 16.09 | 0.47 | 0.29 | 0.25 | **0.34** | 0.12 | -1.09 | -1.78 | -2.01 |
| TGF+ | *Ccl5* | 18.41 | 18.93 | 17.08 | 0.06 | 0.05 | 0.14 | **0.08** | 0.05 | -3.98 | -4.42 | -2.85 |
| S unfra | *Ccl5* | 8.85 | 9.43 | 9.92 | 17.47 | 12.43 | 9.32 | **13.07** | 4.11 | 4.13 | 3.64 | 3.22 |
| B unfra | *Ccl5* | 11.64 | 11.18 | 11.97 | 3.39 | 4.44 | 2.79 | **3.54** | 0.84 | 1.76 | 2.15 | 1.48 |
| S Fibro | *Ccl7* | 9.78 | 9.70 | 9.98 | 1.02 | 1.07 | 0.91 | **1** | 0.08 | 0.03 | 0.10 | -0.14 |
| B Fibro | *Ccl7* | 8.20 | 8.95 | 8.53 | 2.58 | 1.66 | 2.13 | **2.13** | 0.46 | 1.37 | 0.73 | 1.09 |
| Myo | *Ccl7* | 11.01 | 10.99 | 12.10 | 0.49 | 0.50 | 0.26 | **0.42** | 0.14 | -1.02 | -1.00 | -1.94 |
| TGF+ | *Ccl7* | 15.42 | 14.98 | 16.02 | 0.04 | 0.05 | 0.03 | **0.04** | 0.01 | -4.76 | -4.38 | -5.26 |
| S unfra | *Ccl7* | 9.78 | 9.97 | 10.32 | 1.02 | 0.91 | 0.74 | **0.89** | 0.14 | 0.03 | -0.14 | -0.43 |
| B unfra | *Ccl7* | 10.89 | 10.92 | 10.99 | 0.53 | 0.52 | 0.50 | **0.52** | 0.02 | -0.91 | -0.94 | -1.00 |
| S Fibro | *Ccl11* | 8.71 | 7.61 | 7.94 | 0.67 | 1.28 | 1.05 | **1** | 0.31 | -0.58 | 0.36 | 0.08 |
| B Fibro | *Ccl11* | 9.15 | 9.07 | 9.04 | 0.52 | 0.54 | 0.55 | **0.54** | 0.02 | -0.95 | -0.88 | -0.86 |
| Myo | *Ccl11* | 15.38 | 16.02 | 15.72 | 0.01 | 0.01 | 0.01 | **0.01** | 0.00 | -6.24 | -6.78 | -6.52 |
| TGF+ | *Ccl11* | 26.88 | 26.11 | 27.09 | 0.00001 | 0.00002 | 0.00001 | **0.00002** | 0.00001 | -16.09 | -15.33 | -16.09 |
| S unfra | *Ccl11* | 19.15 | 15.69 | 14.52 | 0.001 | 0.01 | 0.02 | **0.01** | 0.01 | -9.43 | -6.50 | -5.51 |
| B unfra | *Ccl11* | 11.24 | 16.16 | 10.65 | 0.15 | 0.01 | 0.21 | **0.12** | 0.11 | -2.72 | -6.89 | -2.22 |
| S Fibro | *Cxcl1* | 10.77 | 10.67 | 10.09 | 0.85 | 0.90 | 1.26 | **1** | 0.23 | -0.24 | -0.16 | 0.33 |
| B Fibro | *Cxcl1* | 9.83 | 10.32 | 10.04 | 1.47 | 1.10 | 1.29 | **1.29** | 0.19 | 0.55 | 0.13 | 0.37 |
| Myo | *Cxcl1* | 12.88 | 14.02 | 13.99 | 0.24 | 0.12 | 0.13 | **0.16** | 0.07 | -2.04 | -3.01 | -2.98 |
| TGF+ | *Cxcl1* | 22.46 | 26.30 | 19.17 | 0.001 | 0.0001 | 0.01 | **0.002** | 0.003 | -10.16 | -13.42 | -7.37 |
| S unfra | *Cxcl1* | 14.62 | 16.18 | 14.31 | 0.09 | 0.03 | 0.11 | **0.08** | 0.04 | -3.51 | -4.84 | -3.25 |
| B unfra | *Cxcl1* | 14.64 | 15.20 | 13.00 | 0.09 | 0.06 | 0.23 | **0.13** | 0.09 | -3.53 | -4.00 | -2.14 |
| S Fibro | *Cxcl2* | 12.72 | 11.89 | 12.68 | 0.82 | 1.34 | 0.84 | **1** | 0.29 | -0.28 | 0.42 | -0.25 |
| B Fibro | *Cxcl2* | 11.06 | 11.49 | 12.02 | 2.19 | 1.70 | 1.24 | **1.71** | 0.47 | 1.13 | 0.77 | 0.31 |
| Myo | *Cxcl2* | 13.57 | 13.64 | 14.05 | 0.50 | 0.48 | 0.38 | **0.45** | 0.07 | -1.00 | -1.06 | -1.41 |
| TGF+ | *Cxcl2* | 22.39 | 27.33 | 19.18 | 0.003 | 0.0002 | 0.02 | **0.01** | 0.01 | -8.48 | -12.39 | -5.76 |
| S unfra | *Cxcl2* | 6.63 | 6.58 | 7.31 | 29.49 | 30.33 | 19.80 | **26.54** | 5.85 | 4.88 | 4.92 | 4.31 |
| B unfra | *Cxcl2* | 8.17 | 8.20 | 7.85 | 11.91 | 11.70 | 14.39 | **12.67** | 1.49 | 3.57 | 3.55 | 3.85 |
| S Fibro | *Cxcl5* | 16.74 | 15.73 | 13.81 | 0.36 | 0.65 | 2.00 | **1** | 0.87 | -1.48 | -0.63 | 1.00 |
| B Fibro | *Cxcl5* | 13.96 | 14.25 | 14.05 | 1.82 | 1.54 | 1.73 | **1.70** | 0.14 | 0.87 | 0.62 | 0.79 |
| Myo | *Cxcl5* | 17.77 | 18.00 | 17.97 | 0.20 | 0.17 | 0.17 | **0.18** | 0.01 | -2.36 | -2.56 | -2.53 |
| TGF+ | *Cxcl5* | 21.40 | 27.00 | 27.00 | 0.02 | 0.001 | 0.001 | **0.01** | 0.01 | -5.44 | -10.19 | -10.19 |
| S unfra | *Cxcl5* | 17.54 | 13.45 | 16.56 | 0.22 | 2.47 | 0.40 | **1.03** | 1.25 | -2.16 | 1.30 | -1.33 |
| B unfra | *Cxcl5* | 15.45 | 16.16 | 16.98 | 0.76 | 0.50 | 0.31 | **0.52** | 0.23 | -0.39 | -1.00 | -1.69 |
| S Fibro | *Cxcl10* | 10.75 | 10.16 | 10.41 | 0.83 | 1.17 | 1.01 | **1** | 0.17 | -0.28 | 0.22 | 0.01 |
| B Fibro | *Cxcl10* | 10.02 | 10.50 | 10.50 | 1.27 | 0.96 | 0.95 | **1.06** | 0.18 | 0.34 | -0.06 | -0.07 |
| Myo | *Cxcl10* | 11.50 | 11.96 | 11.13 | 0.53 | 0.40 | 0.66 | **0.53** | 0.13 | -0.91 | -1.31 | -0.60 |
| TGF+ | *Cxcl10* | 21.42 | 20.00 | 18.98 | 0.002 | 0.004 | 0.01 | **0.004** | 0.003 | -9.33 | -8.12 | -7.26 |
| S unfra | *Cxcl10* | 9.81 | 9.99 | 12.16 | 1.43 | 1.57 | 0.31 | **1.11** | 0.69 | 0.52 | 0.65 | -1.69 |
| B unfra | *Cxcl10* | 13.17 | 13.46 | 13.99 | 0.20 | 0.20 | 0.11 | **0.17** | 0.06 | -2.33 | -2.29 | -3.24 |
| S Fibro | *Cxcl12* | 7.35 | 6.43 | 6.69 | 0.71 | 1.23 | 1.05 | **1** | 0.26 | -0.49 | 0.30 | 0.08 |
| B Fibro | *Cxcl12* | 6.02 | 6.21 | 6.70 | 1.56 | 1.40 | 1.05 | **1.34** | 0.26 | 0.64 | 0.49 | 0.07 |
| Myo | *Cxcl12* | 8.99 | 8.99 | 10.10 | 0.27 | 0.27 | 0.14 | **0.23** | 0.08 | -1.88 | -1.87 | -2.82 |
| TGF+ | *Cxcl12* | 8.34 | 7.64 | 7.00 | 0.40 | 0.60 | 0.88 | **0.63** | 0.24 | -1.32 | -0.73 | -0.18 |
| S unfra | *Cxcl12* | 9.81 | 10.27 | 9.61 | 0.17 | 0.13 | 0.19 | **0.16** | 0.03 | -2.57 | -2.96 | -2.40 |
| B unfra | *Cxcl12* | 12.16 | 11.96 | 11.93 | 0.04 | 0.05 | 0.05 | **0.05** | 0.003 | -4.56 | -4.39 | -4.37 |
| S Fibro | *Cxcl14* | 9.21 | 8.16 | 8.99 | 0.75 | 1.39 | 0.86 | **1** | 0.34 | -0.42 | 0.48 | -0.22 |
| B Fibro | *Cxcl14* | 7.15 | 6.98 | 8.43 | 2.52 | 2.78 | 1.19 | **2.16** | 0.85 | 1.33 | 1.48 | 0.25 |
| Myo | *Cxcl14* | 7.60 | 7.26 | 7.42 | 1.93 | 2.36 | 2.15 | **2.15** | 0.21 | 0.95 | 1.24 | 1.10 |
| TGF+ | *Cxcl14* | 9.45 | 9.44 | 9.41 | 0.65 | 0.66 | 0.67 | **0.66** | 0.01 | -0.62 | -0.61 | -0.59 |
| S unfra | *Cxcl14* | 10.57 | 10.97 | 10.76 | 0.34 | 0.27 | 0.30 | **0.30** | 0.04 | -1.57 | -1.91 | -1.72 |
| B unfra | *Cxcl14* | 10.66 | 10.65 | 10.97 | 0.32 | 0.32 | 0.27 | **0.30** | 0.03 | -1.64 | -1.63 | -1.91 |
| S Fibro | *Cx3cl1* | 14.01 | 13.07 | 15.55 | 0.95 | 1.66 | 0.39 | **1** | 0.64 | -0.07 | 0.73 | -1.37 |
| B Fibro | *Cx3cl1* | 12.14 | 12.41 | 13.04 | 2.87 | 2.44 | 1.69 | **2.33** | 0.60 | 1.52 | 1.29 | 0.76 |
| Myo | *Cx3cl1* | 13.40 | 14.03 | 12.97 | 1.37 | 0.94 | 1.76 | **1.36** | 0.41 | 0.45 | -0.08 | 0.81 |
| TGF+ | *Cx3cl1* | 13.44 | 14.02 | 12.99 | 1.33 | 0.95 | 1.74 | **1.34** | 0.39 | 0.41 | -0.07 | 0.80 |
| S unfra | *Cx3cl1* | 9.68 | 10.00 | 12.03 | 12.15 | 10.08 | 3.06 | **8.43** | 4.77 | 3.60 | 3.33 | 1.61 |
| B unfra | *Cx3cl1* | 13.12 | 13.79 | 10.97 | 1.61 | 1.09 | 5.70 | **2.80** | 2.52 | 0.69 | 0.12 | 2.51 |
